# A systematic approach to analyse the impact of farm-profiles on bovine health

**DOI:** 10.1038/s41598-021-00469-2

**Published:** 2021-10-27

**Authors:** Caspar Matzhold, Jana Lasser, Christa Egger-Danner, Birgit Fuerst-Waltl, Thomas Wittek, Johann Kofler, Franz Steininger, Peter Klimek

**Affiliations:** 1 Section for Science of Complex Systems, Center for Medical Statistics, Informatics, and Intelligent Systems, Medical University of Vienna, 1090 Vienna, Austria; 2 Complexity Science Hub Vienna, Josefstädterstraße 39, 1080 Vienna, Austria; 3 Institute for Interactive Systems and Data Science, Graz University of Technology, Inffeldgasse 16C, 8010 Graz, Austria; 4 ZuchtData EDV-Dienstleistungen GmbH, Dresdner Straße 89/B1/18, 1200 Vienna, Austria; 5 University of Natural Resources and Life Sciences, Gregor-Mendel-Straße 33, 1180 Vienna, Austria; 6 Vetmeduni Vienna, University Clinic for Ruminants, Veterinärplatz 1, 1210 Vienna, Austria

**Keywords:** Statistics, Preventive medicine, Data integration

## Abstract

In this study we present systematic framework to analyse the impact of farm profiles as combinations of environmental conditions and management practices on common diseases in dairy cattle. The data used for this secondary data analysis includes observational data from 166 farms with a total of 5828 dairy cows. Each farm is characterised by features from five categories: husbandry, feeding, environmental conditions, housing, and milking systems. We combine dimension reduction with clustering techniques to identify groups of similar farm attributes, which we refer to as farm profiles. A statistical analysis of the farm profiles and their related disease risks is carried out to study the associations between disease risk, farm membership to a specific cluster as well as variables that characterise a given cluster by means of a multivariate regression model. The disease risks of five different farm profiles arise as the result of complex interactions between environmental conditions and farm management practices. We confirm previously documented relationships between diseases, feeding and husbandry. Furthermore, novel associations between housing and milking systems and specific disorders like lameness and ketosis have been discovered. Our approach contributes to paving a way towards a more holistic and data-driven understanding of bovine health and its risk factors.

## Introduction

Digitisation in dairy cattle farming holds the promise of substantially improving early detection and prevention of disease. Through the collection, linkage, and analysis of routine data generated by milking, feeding, housing and environment, health monitoring and performance recording systems, new disease risk factors can be identified.

Nevertheless, the detection of disease risk factors on the basis of such data is considerably complicated by the heterogeneity of the data itself and, above all, by the fact that diseases are often caused by a combination of multiple factors, rather than individual factors. In order to understand this complexity of disease, knowledge of cow- as well as on farm-related properties and their interactions is required. While the impact of cow-related factors, such as milk yield or body condition score (BCS) on individual disease outcomes has been researched extensively^1–4^, knowledge gaps still exist concerning the relationship between farm management practices and common diseases. In a recent study, Lasser et al.^5^ revealed in a random forest-based analysis that diseases in dairy cattle are often caused by a complex interplay between a large number of areas of life such as housing, management, nutrition or climate. Therefore, the main goal of this study is to provide a systematic approach to analyse disease risks in relation to a comprehensive set of cow-, farm-related and environmental parameters and their interrelations; we refer to this as a farm profile. We combine machine learning techniques with statistical analysis to investigate the relation between these farm profiles and their associated risks for the eight most frequent diseases in our data source; the so-called disease risk profile. Such a risk profile describes the impact of a group of interacting risk factors and other variables on the likelihood of observing a given disease in an animal at a farm with a corresponding farm profile.

Here, we perform an observational study to identify disease risk profiles for different clusters of farms. We aggregate extensive field-based datasets from five different sources, containing 142 variables including environmental factors and information on management, husbandry, housing, milking and feeding; see “Materials”. We proceed in three steps; see “Methods”. First, we apply the uniform manifold approximation and projection for dimension reduction algorithm (UMAP)^6^ to identify pertinent factors by reducing the dimensionality (number of independent variables) and thereby complexity of the aggregated data. Second, we use the hierarchy density-based spatial clustering of applications with noise algorithm (HDBSCAN)^7^ procedure to identify groups (clusters) of farms with similar attributes. Each cluster defines a farm profile as a combination of environmental factors and management practices. A disease risk profile was assessed by comparing disease prevalence between the individual clusters. Third, we applied a multivariate regression model to understand the impact of the individual risk factors on diseases.

## Materials and methods

### Materials

The data used for this analysis includes 166 farms with a total of 5828 cows participating in the research project “Efficient Cow” (Egger-Danner et al.^8^). Each farm is characterised by 142 variables which contain detailed farm-related information along five categories: environmental conditions, husbandry, feeding, housing and milking systems.

#### Husbandry, housing and milking systems

Data about husbandry, housing and milking systems has been collected by a survey within the scope of the “Efficient Cow” project^8,9^. The survey was conducted by trained staff from the performance recording organisations at all 166 farms, assessing information on farm management practices describing the management for young stock, lactating and dry cows. The original farm-based dataset included 70 variables: 8 numeric, 2 nominal and 60 categorical. Variables that contained irrelevant information for the analysis, such as the code for main production area or the unique identifier of hoof trimmers were excluded after consultation with domain experts. For the statistical analysis, categorical variables were one-hot-encoded. Individual categories with less than ten observations were grouped together as a new variable labelled as “other”. Each variable was then tested for internal consistency. A variable was included if it contained at least 80otherwise.

#### Feeding

Information about feed composition and feeding stems from 54,661 cow-based observations of the 5828 animals that were conducted within the scope of routine dairy herd improvement assessments (DHI) between 2/1/2014 and 5/3/2015. To estimate a farm’s feed composition, we calculated the average amount of crude fiber and concentrate feed and their respective standard deviations for lactating and dry cows of a given farm. Information about feed quality stems from feed quality assessments conducted in 2014^10^. These cow-based observations were also matched to diagnoses based on unique and pseudonymised animal identifiers and the observation and diagnosis date, respectively. This data set was then used in the multivariate logistic regression (see “Methods” section “Multivariate logistic regression model” below) to assess the impact of individual features on the odds for specific diseases. The assessors rated the contamination of feed with mold as well as feed temperature as an indication for fermentation on a scale from 1 (perfect) to 4 (unsatisfactory). As assessments returned a perfect rating for both categories in almost all instances, feed quality was defined as problematic when the average of these two ratings was higher than 1.1.

#### Weather

Records from the national weather service from 2014 were used to determine the environmental conditions of a farm. Based on the farms’ locations we computed its yearly average temperature, precipitation and humidity and the corresponding standard deviations. Furthermore, we computed the number of days with high wind and low temperature conditions. A day with at least one measurement smaller than $0.5\,{^\circ {\hbox {C}}}$ was classified as a low temperature day^11^, and a day with at least one observation of wind speed greater than $2.5\,{\hbox {m/s}}$ was classified as a windy day.

#### National disease register

We used information about diagnoses from the national disease register to calculate disease prevalence for the eight most common diseases for each farm. Entries in the national disease registry are either diagnoses by veterinarians^12^ or observations at calving and culling reasons^13^. The main data source for diagnostic information is field data based diagnoses by veterinarians, which must be documented due to legal regulations. This information is predominantly transmitted by the veterinarian directly to the central cattle database (RDV)^12^. Additional health-related observations from farmers around calving are recorded by employees from the performance recording organisation^13^. These employees were trained in advance, before starting the nationwide data collection at these 166 farms, in locomotion scoring, using the Sprecher et al.^14^ method in real farms and using videos showing different locomotion scores. All health-related information is collected according to the law on the control of veterinary medicinal products (Tierarzneimittelkontrollgesetz), which requires a standardization and validation of the data. Health-related information is standardised using a coding system consisting of 65 diagnoses divided into ten categories. The diagnosis code for the Austrian National Disease Register is based on the very extensive ICAR central health code^15^. In addition to plausibility checks, the validation of the data contained is based on a strict criterion for the regular and complete recording of diagnoses. Furthermore, we derived information on diagnoses for lameness and ketosis based on recurring ketosis tests and lameness scoring. A ketosis diagnosis was assigned to a cow if beta-hydroxybutyrate levels (ketones) of $\ge 200 \upmu {\text {mol}}/{\text {l}}$^16^ was measured either 7 or 14 days after calving. Lameness was diagnosed if a cow had a lameness-score equal to or greater than 3^14,17^. In the present analysis we refer to all the described disease labels as “diagnosis”, regardless of their origin.

We aggregated the information contained in all data sets except for the diagnoses based on unique and pseudonymized farm identifiers. Categorical variables were one-hot encoded. The final aggregated data set used to analyse farm profiles contained 119 binary variables and 23 numeric variables.

### Methods

In our methodological approach, we combine machine learning techniques with statistical analysis. To identify farm profiles, we combined the UMAP and HDBSCAN machine learning technique. For the evaluation of the farm profiles and their associated disease risks, we conduct a statistical analysis that includes calculating cluster-wise ORs and z-scores obtained from a multivariate regression model.

#### UMAP dimension reduction algorithm

In order to identify pertinent factors, we first reduced the dimensionality of the aggregated data. We applied the uniform manifold approximation and projection for dimension reduction (UMAP^6^ to investigate the underlying structure in the farm-based dataset. UMAP is an algorithm for dimension reduction based on manifold learning techniques and ideas from topological data analysis, constructed from a theoretical framework based on Riemannian geometry and algebraic topology. UMAP optimizes the layout of the data representation by creating a two-dimensional description of a high-dimensional dataset. Therefore, this approach preserves more of the hidden global structure^6,18^ within the data than other linear dimensionality reduction techniques such as principal component analysis (PCA). Number of neighbours and minimal distance are the main parameters to define. We set number of neighbours to 50 and minimal distance to 0 as suggested in the documentation on using UMAP as a precursor step to clustering^18^. To improve the analysis, we assigned weights to the individual variables. As weights, we used the test statistic obtained by the multivariate logistic regression model; see below. To estimate the overall importance of a given factor, we computed the average of all test statistic results. Factors, for which no test statistics could be obtained because they did not meet our regression analysis criteria, see “Methods”-“Multivariate logistic regression model”, were omitted. They were considered to be less important, and exclusion allows a uniform analysis without special treatment of individual features. A total of 13 variables were excluded this way, reducing the number of features to 129. Nevertheless, the information of these 13 variables is not lost. The features were excluded for the purpose of clustering, but all 142 available variables were later used to characterise the individual farm profiles, based on their z-scores.

#### HDBSCAN clustering algorithm

The two-dimensional embedding of our dataset obtained from the UMAP analysis was then used as input for the HDBSCAN clustering algorithm to identify regions of similar density and therefore assign farms to clusters (groups) with similar attributes. The hierarchy density-based spatial clustering of applications with noise algorithm (HDBSCAN)^7,19^ constructs a density-based cluster hierarchy tree and then uses a specific stability measure to extract flat clusters from the tree. Each obtained cluster defines a farm-profile as a combination of environmental factors, feeding characteristics and management practices. The minimum cluster size has to be defined manually and was set to 15 for the present analysis. The optimal number of clusters is not an input parameter and is identified by the algorithm. Unlike many other clustering algorithms, HDBSCAN can also refrain from assigning an observation to a cluster if it is too uncertain about its belonging. The mathematical procedure to reduce the complexity of a higher dimensional space is based on stochastic computation, whereby the same analysis may produce different results. To ensure that the identification of relevant factors in our data is robust, we applied the UMAP algorithm in conjunction with HDBSCAN over 10,000 times with different random seeds and recorded the number of identified clusters identified. This approach showed a clear preference for five clusters. For the present analysis, we used a UMAP embedding that results in five distinct clusters and where all farms in the dataset are assigned to a cluster.

#### Disease risk profile

A disease risk profile for every cluster was created by comparing disease prevalence between the individual clusters. The disease risk of a profile was examined for the eight most common diseases in our data: anestrous, acute and chronic mastitis, ketosis, lameness, metritis, periparturient hypocalcemia and ovarian cysts. For every disease we identified the cluster with the lowest disease prevalence and used it as the reference group to calculate the OR of that disease for the other clusters. We only used clusters if they had at least five cows diagnosed with a certain disease. The OR was computed using a one-sided Fisher’s exact test.

#### z-Scores

In order to identify the pertinent variables of a farm profile, we computed z-scores for all variables of each cluster. To interpret a z-score on a cluster level, we computed the z-scores in comparison to as follows:

$$\begin{aligned} z= \frac{x -\mu }{\sigma } \end{aligned}$$

where x is the cluster mean value of a variable, $\mu$ the global mean value of all clusters and $\sigma$ the standard deviation of all clusters. Here, a z-score measures how many standard deviations a variable of a given cluster is above or below the global mean. The corresponding p-value determines whether the variable differs significantly from the observed values in the other clusters. Significance was tested using a 2-sided t-test for numeric variables, and a Fisher’s exact test for binary variables. A cluster variable with a high z-score is interpreted as a pertinent factor for that cluster, and the p-value specifies if the variable is significantly different from the other clusters. The obtained cluster specific z-score ranges differ between the clusters. For a better comparison of the factor importance between the clusters, we introduced a ranking of the z-scores from 1 to 142, where the highest z-score is assigned a 1 and the lowest 142.

#### Multivariate logistic regression model

To investigate the impact of the individual factors on diseases, we applied multivariate logistic regression. The dependent variable is the disease, which is explained by independent variables. The analysis was carried out based on the observation dataset linked to the diagnoses. We matched every diagnosis to one out of the 54,661 observations based on animal label and temporal proximity. We only included observations before the occurrence of a diagnosis even if an observation of the same animal after the diagnosis was closer in time, to exclude a possible bias introduced by the farmer’s reaction to a disease. Furthermore, all observations of dry cows were excluded due to their specific treatment. A cow undergoes a metabolic change during dry periods that needs a change in management, such as the management of feeding. Therefore, observations from the dry period might not be representative for the relationship between disease and factor outside of the dry period. Animals with no diagnosis during the entire observation period were considered as the control group. In the logistic regression, 6615 observations linked to a diagnosis were analysed compared to 20,932 observations in the control group. Due to the heterogeneity of the data, numerical variables were normalized to a mean of zero and variance of one. To minimize the effects of confounding variables in the regression model we adjusted for the number of lactations, season, breed, the performance level of the farm (assessed by average yearly milk yield) as well as for differences in the diagnoses’ origins (veterinarian, observation close to calving/culling reason or based on a keto-test or lameness scoring). We corrected for biases due to different reporting schemes in certain regions by introducing a binary variable that indicates whether the diagnosis was made within such a region or not. In the regression model, the association between a given independent variable and a disease was estimated by keeping the other (confounding) variables constant. For each variable–disease pair with more than 50 observations, we applied the logistic regression model. To correct for multiple hypothesis testing, we applied the Benjamini-Hochberg procedure. The variance inflation factor was computed to control for possible multicollinearity. To improve interpretability of the obtained results, we converted the log odds into odds ratios (OR). The odds ratios were used to investigate the effects of each factor on diseases, particularly those factors characteristic of a farm profile.

## Results

We report our results in the following structure. First, we describe the five farm profiles obtained by dimension reduction and clustering. Second, we describe and discuss the obtained disease risk profiles for these farm profiles. We conclude the manuscript by discussing technical limitations of our approach and summarizing our main findings. Statistical significance will be reported as *** for $p<0.001$, ** for $p<0.01$ and * for $p<0.05$.

### UMAP-HDBSCAN-clustering

**Figure 1:**
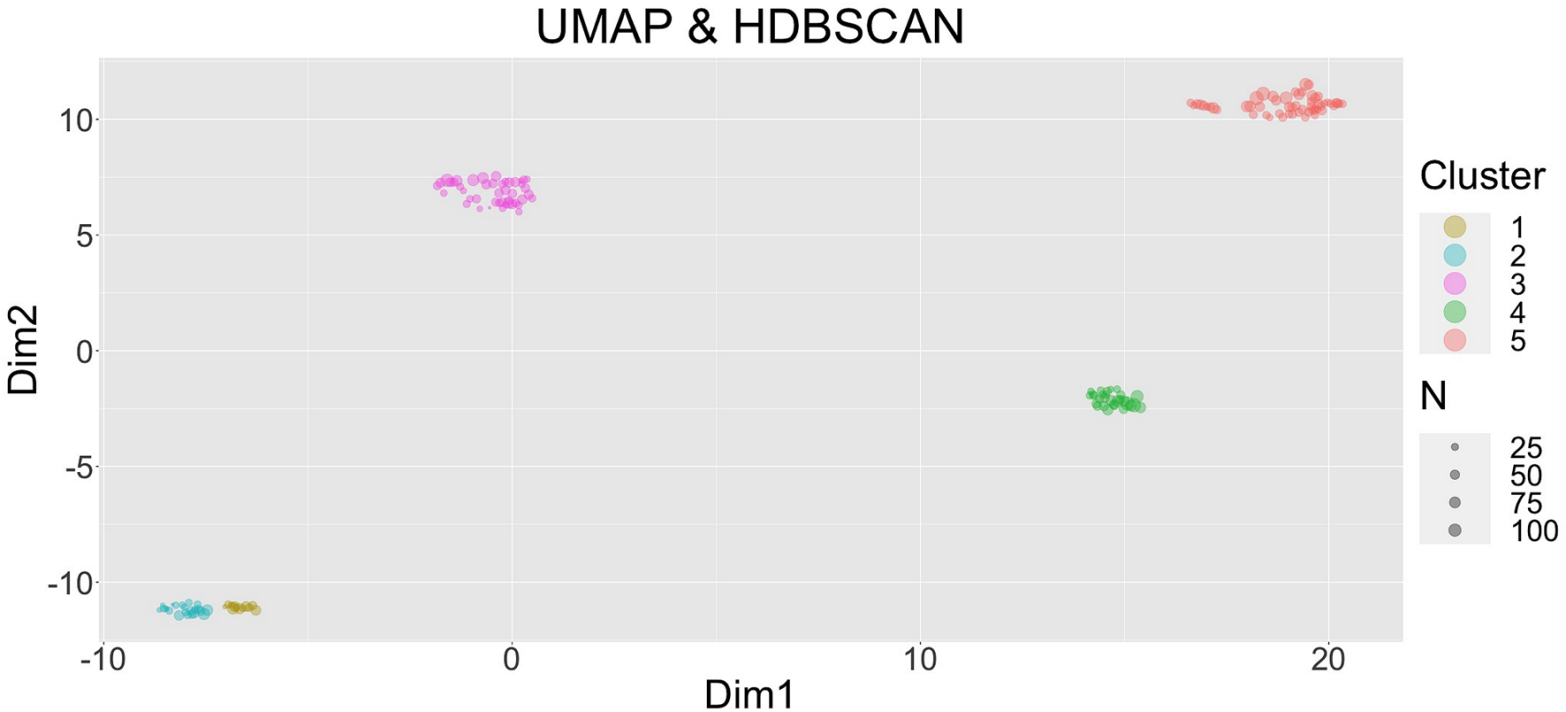
UMAP-HDBSCAN clustering. Each circle corresponds to a farm and the size of the circle is proportional to number of cows on a farm. Five clusters of farms can be identified, which are represented by different colours.

The obtained results of the UMAP-HDBSCAN cluster analysis reveal five well-separated clusters of farms, the farm profiles; see Fig. 1. The farms have been grouped along the two-dimensional representation of our data as inferred with the UMAP procedure. Each dot represents one of 166 farms, and the size is proportional to the number of cows. For descriptive statistics for all variables see Supplement Table 6. In the following, we present the pertinent factors that characterise a farm profile structured along five categories: environmental conditions, husbandry, feeding, as well as housing and milking systems.

#### Farm profile of cluster 1

Cluster 1 describes the farm management of 12 farms located at a high altitude (i.e., height above mean sea level) [z-score-rank: 2*** p-value < 0.001] that are exposed to a high number of days with low temperature [rank 3***]. The farm management of this cluster is characterised by providing access to alpine pasture and pasture. We observe high ranks for providing access to alpine pasture for young stock [rank 8** p-value < 0.01], lactating [rank 16] and dry cows [rank 5***] as well as for pasture for young stock [rank 24], lactating [rank 35] and dry cows [rank 11* p-value < 0.05]. With regard to husbandry, we note that claw trimming is performed twice a year [rank 13*]. Cows of this cluster tend to be kept in a free-stall barn [rank 10**]. In terms of housing, we find that rubber mats are used to cover the walkway in free-stalls for dry cows [rank 7*]. Cows are either milked by a milking robot system [rank 18], or a side-by-side milking system [rank 22], which are equipped with a milking unit removal system including post-milking technology [rank 20]. In addition to pasture, grass silage [rank 9**] is used for feeding.

#### Farm profile of cluster 2

Similar to cluster 1, cluster 2 describes the management of farms at high altitude [rank 1***] that are exposed to low temperatures [rank 9***]. In total, cluster 2 contains 23 farms. Cows of this cluster have access to alpine pasture, but less to pasture when compared to cluster 1. We find high ranks in providing access to the alpine pasture for young stock [rank 2***] and dry cows [rank 4***], while for access to pasture only for young stock [rank 14**] a high rank is observed. In comparison to cluster 1, we observe within cluster 2 indicators for free- and tie-stall housing management. We observe that young stock [rank 22*] and lactating cows are kept in tie-stall facilities [rank 12**]. Some farms, on the other hand, tend to hold lactating cows [rank 16*] in a free-stall system with deep bed cubicles. Cows of this cluster are milked by a pipe milking system [rank 21*], including a milking vacuum [rank 18***] without a milking unit removal system [rank 27*]. The feeding management is characterised by the use of silage bales [rank 6**] and a feed consisting of grass, grass products and corn [rank 8**]. Therefore, the mean content of crude fibre in the fodder for dry [rank 25*] and lactating cows [rank 3***] is high. A negative z-value in herd size [rank 13**] indicates small farms.

#### Farm profile of cluster 3

Cluster 3 also describes the management of farms at high altitude [rank 10***]. In contrast to cluster 1 and 2, the 45 farms in this cluster are exposed to high annual precipitation [rank 2**] and a high deviation in annual precipitation [rank 14]. Regarding pasture management, we find indicators that most farms in this cluster do not have access to alpine pasture. For pasture we only observe a high rank in lactating cows [rank 9*]. As in cluster 2, we find in cluster 3 indicators for free- and tie-stall housing management. The minority of farms of cluster 3 keep cows in a tie-stall facility during lactation [rank 16] and the dry period [rank 18]. The majority of farms of cluster 3, however use a free-stall system with deep bed cubicles in combination with a scraper for manure removal. We observe the combination of deep bed cubicles and scraper for dry and lactating cows [rank 4*,19; rank 17,6*]. Cows of this cluster tend to be milked using a pipe milking system [rank 5**] in combination with a milking unit removal [rank 7*]. Regarding feeding management, we find a trend towards a grass-based diet. Farms in this cluster feed mostly grass and grass products [rank 8*]. In addition, we observe that no silos are used for feed storage [rank 3**].

#### Farm profile of cluster 4

In cluster 4 we find indicators for a milk intensive farm management [rank 19*] and large herds [rank 31]. The 29 farms in this cluster are exposed to a comparatively high yearly temperature [rank 4***] with a high standard deviation [rank 3***], a high relative humidity [rank 53**] and many hot [rank 2***] and windy days [rank 7***]. Due to the environmental conditions the most common barn design of this cluster is an outdoor climate housing system with an open front [rank 29]. Cows tend to be kept in a free-stall system with walkways with concrete slits, indicated by a high rank of this feature for young stock [rank 18*]. The housing is further characterised by the use of high bed cubicles for young stock [rank 22*]. In terms of milking systems, no prioritised system can be identified, but we find a frequent use of a milking unit removal including a post-milking technology [rank 24*]. Regarding feeding management, we observe a tendency toward less individualized feeding indicated by the use of a total mixed ration (TMR). This method is used in the distribution of forage [rank 34] as well as of concentrate [rank 38]. The feeding of this cluster is characterised by a year-round feeding with corn silage [rank 12***] and a diet which is composed of field forage silage, grass silage, hay and corn silage [rank 14**]. A high dietary proportion of concentrates in lactating cows is observed [rank 26**]. In addition, we find the use of a bunker silo system [rank 16**] within this cluster.

#### Farm profile of cluster 5

Similar to cluster 4, cluster 5 describes the management of rather large herds [27]. Farms of this cluster are also exposed to high annual temperatures [rank 3***] with a high standard deviation [rank 19*], and a high number of hot days [rank 5***]. With a total of 57, most farms in our analyses were assigned to this cluster. Cows of cluster 5 tend to be kept in a closed barn [rank 38]. As cubicle housing system we observe deep bed cubicles with slatted floors [rank 29] as well as high bed cubicles with solid floors [rank 33]. For dry cows we find that high bed cubicles [rank 21] and deep litter [rank 25] are used. For milking we find herringbone parlour [rank 32] as the main milking system. The diet consists of field forage silage, grass silage, hay, corn silage and pasture [rank 8**]. However, in contrast to cluster 4, where the TMR method is used, farms in cluster 5 use a partial mixed ration method (AGR) for the distribution of forage [rank 28] or a mixed forage ration with concentrates [rank 26]. In addition, we observe a high proportion of concentrates [rank 14*] along with a high standard deviation of concentrates [rank 36] in dry cows. In cluster 5 we also find an indicator for problematic feed quality [rank 43].

### Disease risk profiles: grouped feature–disease relations

**Figure 2:**
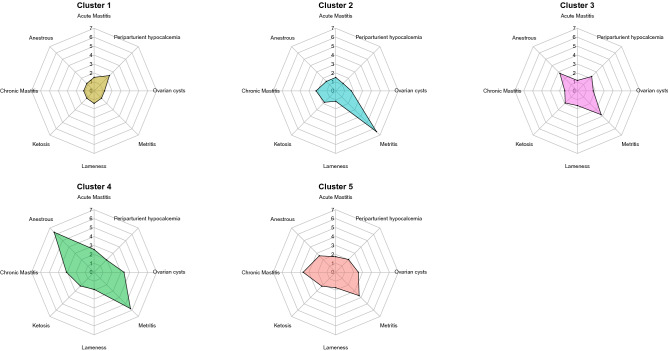
Farm risk profiles. Comparison of the disease frequency in the five clusters. For a given disease, the cluster with the lowest disease prevalence is used as the reference group to calculate the OR of the other clusters.

Here, we report associations between groups of interacting variables comprising the farm profiles and disease risks. As shown in Fig. 2 and Table 1, we find that the observed differences in farm profiles associate with substantial differences in disease risks. In general, we observe the trend that farms in cluster 1 and 2 are associated with a decreased disease risk, while farms of cluster 4 and 5 have an increased disease risk. In cluster 3, we observe a trend towards a moderately increased odds ratios (OR) for metritis [OR: 3.23**], anestrous [OR: 2.38***], periparturient hypocalcemia [OR: 1.95***] and the lowest prevalence of acute mastitis diagnoses as well as the second lowest odds for chronic mastitis. The greatest differences in disease risk are observed between cluster 1 and 4. The farm profile of cluster 4 is associated with the overall highest disease risks, with highest odds ratios for anestrous [OR: 5.43***], lameness [OR: 1.64***], acute mastitis [OR: 2.18***] and ovarian cysts [OR: 2.87***]. In contrast to that, cluster 1 describes a farm profile that has a positive effect on the health and well-being of dairy cattle. Consequently, cluster 1 has the lowest prevalence for ovarian cysts, metritis, chronic mastitis, ketosis and anestrous diagnoses. However, we also observe that cluster 1 has the highest risk for periparturient hypocalcemia [OR: 2.08**]. Further, we observe that cluster 2 has the lowest prevalence of periparturient hypocalcemia and lameness, but also the highest odds ratio for metritis [OR: 5.53***]. In cluster 5 we observe the highest odds for chronic mastitis [OR: 3.13***] and ketosis [OR: 1.89***].

**Table 1:** Odds ratio (OR) of the individual farm risk profiles. Empty fields indicate cluster with the lowest disease prevalence.

Diagnosis	Cluster 1	Cluster 2	Cluster 3	Cluster 4	Cluster 5
Acute mastitis	1.3	1.29		2.18***	1.49***
Anestrous		1.28	2.37***	5.43***	2.23***
Chronic mastitis		1.89	1.21	2.65**	3.13***
Ketosis		1.55*	1.65*	1.87**	1.89***
Lameness	1.2		1.41**	1.64***	1.48***
Metritis		5.53***	3.23**	4.93***	3.17**
Ovarian cysts		1.5	1.53*	2.87***	2.17***
Periparturient hypocalcemia	2.08**		1.95***	1.68**	1.73**

### Multivariate logistic regression: feature–disease relations

For a better understanding of the relationship between diseases and the individual factors, we conducted a multivariate logistic regression analysis. In the following we use the estimates obtained by the logistic regression model to investigate the relationship between individual factors and diseases. The results of the logistic regression model are reported in the “Supplement” in Tables 7–14. Each cluster is analysed based on the factors highlighted in “Results” and their relationship to disease risks identified in “Conclusions”. In the disease risk profiles of cluster 1 and cluster 4, we focus on the observed trends, while we concentrate on the most or least frequently observed diseases in the other profiles. Further, we will focus on effects on lactating cows unless we do not observe any results for them, in which case we report results for dry cows or young stock, if available. We also report results from the latter whenever they differ from results obtained for lactating cows.

#### Disease risk profile for cluster 1

Cluster 1 is, overall, the cluster with the healthiest animals compared to all other clusters, thus showing the lowest disease risk for anestrous, ketosis, chronic mastitis, metritis and ovarian cysts. In this cluster, we observe strong negative correlations between disease risks and environmental conditions and husbandry, respectively. Regarding environmental conditions we find a strong negative association between farms located at a high altitude [rank 2] and all considered diseases, with ORs and confidence intervals (CI) ranging from 0.34 [OR 0.34 CI 0.31–0.38] for anestrous to 0.75 [CI 0.65–0.86] for periparturient hypocalcemia. A high amount of low temperature days [rank 3] reduces the ORs for anestrous, metritis, ovarian cysts, acute mastitis and lameness, with ORs ranging from 0.61 [CI 0.54–0.68] for anestrous to 0.88 [CI 0.84–0.92] for lameness. In terms of husbandry, we observe strong negative effects for alpine pasture and pasture. Farms providing access to pasture [rank 35] show a decreased disease risk for all diseases except for metritis, with ORs ranging from 0.38 [CI 0.28–0.52] for chronic mastitis to 0.72 [CI 0.60–0.86] for acute mastitis. A similar trend is found for a pasture management that provides access to alpine pasture. Alpine pasture in young stock [rank 8] reduces the risk for anestrous, ketosis, metrits and ovarian cysts [OR 0.21 CI 0.17–0.26; OR 0.25 CI 0.18–0.34; OR 0.30 CI 0.22–0.43, OR 0.29 CI 0.24–0.36] and in dry cows [rank 5] for lameness [OR 0.53 CI 0.45–0.63]. For claw trimming that is performed twice a year we observe reduced odds for anestrous, metrits, ovarian cysts and acute mastitis, with ORs ranging from 0.55 [CI 0.42–0.73] for metritis to 0.79 [CI 0.67–0.92] for acute mastitis. Regarding barn design, we observe that a free-stall barn design [rank 10] decreases the risk for anestrous and acute mastitis [OR 0.65 CI 0.55–0.78; OR 0.80 CI 0.69–0.94], while increasing the risk for metritis and lameness [OR 1.57 CI 1.20–2.05; OR 1.24 CI 1.15–1.35]. In terms of housing, we find that rubber mats when used to cover the walkway [rank 28] correlate with ovarian cysts and acute and chronic mastitis, with the strongest correlation for chronic mastitis [OR 1.96 CI 1.49–2.57]. In the case of lameness, we observe a weak positive association in lactating cows [OR 1.14 CI 1.02–1.27] and a negative one in dry cows [rank 7] for the use of rubber mats [OR 0.84 CI 0.72–0.97]. Regarding the milking system, we observe that the use of a milking robot [rank 18] is associated with a lower occurrence of acute mastitis and lameness [OR 0.52 CI 0.40–0.67; OR 0.72 CI 0.64–0.82]. On the other hand, a milking unit removal system including post-milking technology [rank 20] increases the ORs for lameness and acute and chronic mastitis and ketosis [OR 1.53 CI 1.36–1.74; OR 1.82 CI 1.48–2.23; OR 3.30 CI 2.52–4.32; OR 2.12 CI 1.56–2.88]. Regarding feeding, we find that feeding of silage [rank 9] is associated with decreased odds for ovarian cysts, anestrous, lameness, acute mastitis and metritis, with ORs ranging from 0.46 [CI 0.36–0.58] for ovarian cysts to 0.59 [CI 0.42–0.82] for metritis.

The effects of the environmental conditions and husbandry confirms the observed trend towards a general decreased disease risk in cluster 1, in particular for ovarian cysts, metritis, chronic mastitis, ketosis and anoestrous. However, the increased risk of periparturient hypocalcemia in cluster 1 can only be partially explained. We find that a high standard deviation for proportion of concentrates for dry cows [rank 38] is related to increased OR for periparturient hypocalcemia [OR 1.25 CI 1.10–1.42].

#### Disease risk profile for cluster 2

For the farm-profile of cluster 2, which has the lowest prevalence of periparturient hypocalcemia and lameness diagnoses and the highest risk for metritis, we also observe a decreasing effect of high altitude [rank 1], low annual temperature [rank 9] and a husbandry that does provide access to alpine pasture. We find that altitude reduces the ORs for lameness, periparturient hypocalcemia and, contrary to the observed disease risk at a cluster level, also of metritis [OR 0.70 CI 0.67–0.73, OR 0.75 CI 0.65–0.86 OR 0.52 CI 0.45–0.61]. A high number of low temperature days further reduces the risk of metritis and lameness OR 0.62 CI 0.52–0.75 OR 0.88 CI 0.84–0.92]. For the husbandry of this cluster, we observe a negative association between lameness and periparturient hypocalcemia and alpine pasture in young stock [rank 2] [0.57 CI 0.52–0.62, OR 0.31 CI 0.22–0.43] and for lameness also in dry cows [rank 4] [OR 0.53 CI 0.45–0.63]. A negative correlation is also observed between providing access to pasture for young stock [rank 14] and all three diseases: periparturient hypocalcemia, lameness and metritis [OR 0.31 CI 0.22–043, 0.57 CI 0.52–0.62, OR 0.30 CI 0.22–0.41]. Besides the environmental conditions and the husbandry of this cluster, we find indicators that the housing systems of this cluster are overall associated with reduced disease risks. A free-stall system with deep bed cubicles [rank 16] reduces the OR for lameness [OR 0.40 CI 0.36–0.44]. For a housing management where cows are held in a tie stall facility [rank 12] we find a negative association with lameness [OR 0.69 CI 0.58–0.82]. With regard to milking, we find that the use of a pipe milking system [rank 21] reduces the OR for lameness [OR 0.43 CI 0.35–0.53], as does using a higher milking vacuum [rank 18] [ OR 0.86 CI 0.83–0.90]. Regarding feed, we find that a high average content of crude fibre in lactating cows [rank 3] is associated with decreased odds of all eight diseases except for chronic mastitis. Negative associations are found for periparturient hypocalcemia and lameness [OR 0.71 CI 0.60–0.83, OR 0.74 CI 0.71–0.78]. In addition, the use of silage bales [rank 6] reduces the OR for lameness [OR 0.53 CI 0.47–0.60].

#### Disease risk profile for cluster 3

Compared to clusters 1 and 2, in cluster 3 we observe a general trend towards increased disease risk, although this cluster shares the risk lowering effects of altitude and pasturing. Nevertheless, the cluster has the lowest risk for acute mastitis and the second lowest for chronic mastitis. In terms of environmental conditions, we find a negative association between acute and chronic mastitis and altitude [rank 10] [OR 0.57 CI 0.52–0.63, OR 0.63 CI 0.54–0.72]. The same trend is observed for a high annual precipitation [rank 2] [OR 0.57 CI 0.51–0.63, OR 0.43 CI 0.36–0.51]. Regarding husbandry, we observe a negative correlation between providing access to pasture for lactating cows [rank 9] and acute and chronic mastitis [OR 0.72 CI 0.60–9.86, OR 0.38 CI 0.28–0.52]. For the housing of this cluster, we observe that a free-stall system with deep bed cubicles [rank 17] negatively correlates with acute and chronic mastitis [OR 0.62 CI 0.51–0.75, OR 0.30 CI 0.23–0.38]. For the use of a scraper for manure removal, we observe decreased ORs for acute mastitis in dry cows [rank 19] [OR 0.74 CI 0.60–0.91, OR 0.83 CI 0.71–0.97], but increased ORs for chronic mastitis in lactating [rank 6] and dry cows [OR 1.49 CI 1.17–1.89, OR 1.74 CI 1.36–2.24]. In terms of milking, we find that the use of a milking unit removal [rank 7] is related to a reduced risk for acute mastitis [OR 0.83 CI 0.71–0.97]. The low risk for acute mastitis is further indicated by the grazing-based diet of this cluster. Besides providing access to pasture, farms in this cluster tend to feed only grass and grass products [rank 8], which decreases the occurrence of acute mastitis [OR 0.50 CI 0.17–0.26].

#### Disease risk profile for cluster 4

In contrast to the first three clusters, cluster 4 shows the overall highest risk for diseases. Within this cluster we find the highest cluster-based disease risk for anestrous, lameness, acute mastitis and ovarian cysts. This trend towards increased disease risk is again related to the environmental conditions and husbandry of this cluster. In terms of environmental conditions, we find that higher temperatures correlate with disease risks. For instance, a high annual temperature [rank 4] correlates with all diseases except periparturient hypocalcemia, with ORs ranging from 2.99 [CI 2.60–3.43] for anestrous to 1.27 [CI 1.21–1.33] for lameness. A similar trend is found for the number of high temperature days [rank 2], with ORs ranging from 2.56 [CI 2.30–2.84] for anestrous to 1.33 [CI 1.28–1.39] for lameness. In addition, we observe that a high number of windy days [rank 7] moderately correlates with all diseases except chronic mastitis and metritis, with the strongest effect for ketosis [OR 1.48 CI 1.33–1.64]. For the husbandry of this cluster, we observe that a large herd size moderately [rank 31] correlates with all diseases, except periparturient hypocalcemia, with ORs ranging from 1.18 [CI 1.13–1.23] for lameness to 1.59 [CI 1.42–1.78] for chronic mastitis. The main barn design of cluster 4, an outdoor climate house with open front [rank 29], is associated with increased odds for anestrous, acute mastitis and lameness [OR 3.20, CI 2.68–3.82; OR 1.73, CI 1.45–2.06; OR 1.32, CI 1.19–1.47]. In terms of housing management, we find a correlation between the use of high bed cubicles for young stock [rank 22] and acute mastitis, lameness and ovarian cysts [OR 1.88 CI 1.60–2.21, OR 1.65 CI 1.51–1.80, OR 1.46 CI 1.22–1.74]. Regarding milking, we observe that a milking unit removal with a post-milking technology [rank 24] is associated with chronic and acute mastitis, ketosis, and lameness, with highest ORs ranging from 3.30 [CI 2.52–4.32] for chronic mastitis to 1.53 [CI 1.36–1.74] for lameness. Regarding feeding management, we find that the use of a bunker silo [rank 16] is strongly associated with all diseases, except periparturient hypocalcemia, with ORs ranging from 5.82 [CI 2.59–9.43] for chronic mastitis to 1.82 [CI 1.65–2.02] for lameness. Further, we observe that a year-round feeding with corn silage [rank 12] is associated with increased odds for ovarian cysts, chronic mastitis, periparturient hypocalcemia, ketosis and lameness, with ORs ranging from 2.29 [CI 1.90–2.78] for ovarian cysts to 1.42 [CI 1.27–1.59] for lameness. The association between corn silage and disease risks is further indicated by a diet which is composed of field forage silage, grass silage, hay and corn silage [rank 14]. This forage composition correlates with all diseases except, metritis and periparturient hypocalcemia, with ORs ranging from 2.33 [CI 1.77–3.07] for ketosis to 1.15 [CI 1.03–1.28] for lameness. In addition, we note that the TMR feeding method is associated with increased odds for a range of diseases: A TMR forage [rank 34] correlates with ketosis, acute mastitis and lameness [OR 4.83 CI 3.56–6.55; OR 3.22 CI 2.58–4.03; OR 2.59 CI 2.25–2.99]. The same trend is observed when using TMR for the provision of concentrates [rank 38] [OR 5.04 CI 3.72–6.81; OR 3.37 CI 2.70–4.20; OR 2.55 CI 2.21–2.94].

#### Disease risk profile for cluster 5

Similar to cluster 4, Cluster 5 shows a trend towards increased disease risks, with the highest risk for chronic mastitis and ketosis. In terms of environmental conditions, we observe that a high number of hot days [rank 2] correlates with ketosis and chronic mastitis [OR 1.43 CI 1.25–1.64, OR 1.89 CI 1.62–2.20] as well as a high annual temperature [rank 1] [OR 1.32 CI 1.14–1.53, 1.60 CI 1.35–1.89]. A high yearly relative humidity [rank 19] is also associated with increased odds for chronic mastitis and ketosis [OR 1.65 CI 1.41–1.92, OR 1.40 CI 1.21–1.62]. Regarding housing, we observe that a barn design with a closed barn [rank 17] correlates with chronic and acute mastitis [OR 2.92 CI 2.29–3.73, OR 1.22 CI 1.03–1.46]. A free-stall system with high bed cubicles [rank 18] increases the OR for acute mastitis [OR 1.51 CI 1.16–1.98]. Deep bed cubicles with slatted floors [rank 11] correlate with chronic mastitis and ovarian cysts [OR 2.07 CI 1.59–2.69; OR 1.29 CI 1.15–1.69], while decreasing the ORs for lameness and anestrous [OR 0.89 CI 0.81–0.99; OR 0.77 CI 0.63–0.94]. For the main milking system of this cluster, we observe that a herringbone parlour [rank 12] is associated with increased odds for all eight diseases, such as for chronic mastitis and ketosis [OR 2.69 CI 2.09–3.45, OR 1.55 CI 1.22–1.98]. Regarding feeding, we find that a high standard deviation for dietary proportion of concentrate in the feed of dry cows [rank 15] is associated with an overall increased disease risk such as for ketosis and chronic mastitis [OR 1.47 CI 1.31–1.65, OR 1.72 CI 1.54–1.93]. Contrary to cluster 4, we find a partial mixed ration feeding for the distribution of forage. A partial mixed ration (AGR) feeding when used for forage [rank 28] decreases the OR for acute mastitis [OR 0.56 CI 0.42–0.74], but a mixed forage ration with concentrates [rank 26] increases the OR for acute mastitis [OR 1.19 CI 1.02–1.39]. Furthermore, we observe that our indicator for problematic feed quality [rank 43] moderately correlates with acute and chronic mastitis, lameness, ketosis and anestrous, with highest OR for chronic mastitis [OR 1.55 CI 1.41–1.71].

### Discussion

The presented systematic methodological approach demonstrates how to identify and assess a variety of interacting farm management and environmental factors in relation to certain diseases. The obtained results allow to analyse disease risks in relation to a set of potentially interacting factors.

We find that the farm profile of cluster 1 is associated with an overall decreased disease risk. The profile describes the management of farms at high altitude in which cows are often exposed to low temperatures. The overall decreased disease risk in this cluster is further related to the following factors: a free-stall barn, a husbandry that provides access to alpine pasture and pasture, and a feeding management where silo bales are used. In line with the literature, we find that a free-stall system is associated with better udder health^20,21^, lower risk of ketosis and better fertility^22^. Access to pasture and alpine pasture indicate a farm management that allows regular physical exercise. Previous studies revealed that the combination of loose-housing and regular outdoor exercise^21,23^ have positive effects on the health and welfare of cattle. Alpine pasture in particular is beneficial for the fitness of an animal and thus for its longevity^24^. However, there is also evidence that neither a free-stall system nor increased physical exercise is inherently beneficial to dairy cattle’s health^25^. For instance, increased physical activity has been identified as a risk factor for lameness^26,27^.

In cluster 2 we observe that the positive effect of a free-stall system also depends on environmental conditions, the type of floor type and its quality and farm management practices^28^. We find that tie-stalls that allow outdoor access are known to be associated with a reduced risk of lameness^21^. On the other hand, in line with the literature we observe that a free-stall system with deep-bed cubicles is also associated with reduced risk for injuries, in particular for lameness^29–32^. These inconsistent reports on free- and tie-stall housing emphasize the need for further analyses on the disease risks associated with different forms of housing; namely, an analysis that takes into account additional characteristics of the farm profile such as pasture management and environmental conditions. In this context, we find that access to alpine pasture has a positive effect on the fitness of an animal^24^, as does pasturing on claw health, depending on the duration of pasturing^33^. In line with previous reports where periods of heat stress were associated with an increase in the rate of claw horn lesion^34,35^, we find that low temperatures reduce the risk for lameness.

The overall highest diseases risk is associated with the farm profile of cluster 4. The highest risk increases are observed for anestrous, lameness, acute mastitis and ovarian cysts. As the main risk factors of this cluster, we find a combination of high annual temperature and its standard deviation, a husbandry of a large herd size where cows are kept in an outdoor climate barn, a free-stall housing system with high bed cubicles and a walkway with concrete slits, a milking system with post-milking technology and a feeding management that uses bunker silos together with a year-round corn silage feeding and TMR for forage and provision of concentrates. In line with literature, we observe an association between high temperature and an increased risk of illness. Heat stress has been associated with increased standing time^36^, which increases the risk of lameness^26,27,34^. The risk for lameness also correlates with the size of the herd^1,37^. As described, we also observe that an increased risk for disease correlates with housing factors such as high bed cubicles^30^, or a milking system with post-milking technology. In accordance with the literature, we find that feeding corn silage increases lameness^38–40^, and that a TMR feeding method has a strong negative impact on dairy cattle health^25^. This negative association is also illustrated when compared to the farm-profile of cluster 5. Although the profile of cluster 5 is similar to that of cluster 4, cluster 5 shows a reduced risk of disease. In addition to the annual milk yield, we have identified the feeding method as a distinguishing feature between the two clusters. In cluster 5 a partial mixed ration method is used for feeding, while in cluster 4 a TMR method is used. Furthermore, we observe in cluster 5 that the environmental conditions indicate a problematic feed quality. High humidity and high temperature promote fungal growth and a possible toxic contamination of the feed^41,42^. Silage in particular is known as a source of mycotoxigenic fungi^43^. When animals are fed mycotoxin-contaminated diets, toxic effects such as decreased feed intake and milk production or even death can occur^42–44^.

For some individual disease risk associations as in cluster 3, however, we could not find any confirmation from the literature. This is particularly the case for milking-system-related risk factors. We only found one study^45^ that linked herringbone milking parlors to a higher risk of hock lesion, a sign of lameness, than tandem parlors, which is consistent with our findings on lameness risk variables (see “Supplement”).

### Limitations

Due to the observational study design and its secondary data use, our reported findings have an exploratory character. While the diagnosis information was collected by trained personnel according to clearly defined and strict guidelines, as prescribed by Austrian law, our data comes with the usual limitations of field-based observations. The identified associations therefore need to be evaluated by further research and prospective studies, especially for those of our findings that have not yet been thoroughly addressed in the literature. Care has to be taken when generalising from our findings for Austria to other countries due to unique geographic characteristics of farming in Austria. We found that altitude has a strong impact on the results of the clustering. Altitude and the associated environmental conditions featured prominently amongst the most important variables for defining the clusters. We, therefore, tested the dimension reduction clustering procedure with and without altitude. We observed that without including altitude as a variable, a significant number of farms would have remained unclassified. However, it became clear that the outcomes concerning other variables than environmental ones did not change qualitatively when comparing the farm risk profiles with and without altitude. In other words, associations between farm management practices and diseases have shown to be robust with respect to the in- or exclusion of environmental variables.

## Conclusions

In this study, we presented a systematic framework to analyse the impact of farm profiles as combinations of environmental conditions and management practices on common diseases in dairy cattle. Our findings show that analysing disease risks in relation to a collection of variables can lead to a more holistic description of relevant risk factors and their interrelations as opposed to analysing disease risk in relation to individual variables, which may lead to seemingly inconsistent results if taken out of the context of other farm variables, as has been shown for the free- and tie-stall effects described in “Disease risk profile for cluster 2”. In addition, our analysis reveals new relationships between housing and milking systems and specific diseases. Further prospective studies are needed to examine these relationships in more detail.

## Supplementary information

Supplementary Tables.
