# Supplementary material for: A systematic approach to analyse the impact of farm-profiles on bovine health
Source: Sci Rep. 2021 Oct 27;11:21152. doi: 10.1038/s41598-021-00469-2 (PMC8551198; doi:10.1038/s41598-021-00469-2)
Supplement: Supplementary file 1 — Supplementary Tables. [file 41598_2021_469_MOESM1_ESM.pdf]

Supplement: A systematic approach to analyse the impact of farm-profiles on bovine health

Table 1. Cluster 1 z-score. Scores are ranked according to the absolute value, the highest value is assigned rank 1 and the lowest rank 142.

Variable	Category	Z-Score-Rank	Z-Score	P-Value
mean yearly temperature	environment	1	-2.270	0.00
altitude	environment	2	2.254	0.00
number of low temperature days	environment	3	2.132	0.00
number of high temperature days	environment	4	-1.953	0.00
dry cows on alpine pasture	husbandry	5	1.326	0.00
standard deviation of yearly temperature	environment	6	-1.227	0.00
walkway floor in free-stall for dry cows: rubber mats	housing	7	0.938	0.01
young stock on alpine pasture	husbandry	8	0.902	0.00
silo type: silage bales	feed	9	0.839	0.01
barn design: free-stall barn	housing	10	0.831	0.00
pasture of dry cows	husbandry	11	0.732	0.01
silo type: bunker silo	feed	12	-0.710	0.02
claw trimming frequency: twice per year	husbandry	13	0.662	0.03
manure removal in free-stall for young stock: scraper	housing	14	0.635	0.07
free-stall system for dry cows: other	housing	15	0.619	0.06
lactating cows on alpine pasture	husbandry	16	0.594	0.07
barn design: outdoor climate house closed	housing	17	-0.571	0.04
type of milking stalls: milking robot	milking	18	0.563	0.08
milking unit removal: none	milking	19	-0.562	0.06
milking unit removal: present, including post-milking technology	milking	20	0.519	0.09
manure removal in free-stall for dry cows: slits	housing	21	-0.493	0.16
type of milking stalls: side-by-side	milking	22	0.488	0.11
floor in open-air areas for lactating cows: other	housing	23	0.485	0.12
pasture of young stock	husbandry	24	0.484	0.11
standard deviation content of crude fibre for lactating cows	feed	25	-0.472	0.02
mean content of crude fibre for dry cows	feed	26	0.459	0.19
barn design: outdoor climate house open front	housing	27	-0.459	0.13
walkway floor in free-stall for lactating cows: rubber mats	housing	28	0.448	0.16
floor in walkway of free-stall for young stock: concrete slits	housing	29	-0.447	0.30
used forage types in diet: grass silage, green forage, hay, pasture	feed	30	0.438	0.16
free-stall system for dry cows: high bed cubicle	housing	31	-0.436	0.37
claw trimming done by farmer	husbandry	32	0.435	0.18
free-stall system for lactating cows: other	housing	33	0.434	0.19
cubicle housing system: deep bed cubicles and solid floors	housing	34	0.430	0.14
pasture of lactating cows	husbandry	35	0.419	0.21
floor in open-air area for young stock: unpaved	housing	36	0.412	0.19
free-stall system for lactating cows: high bed cubicle	housing	37	-0.405	0.36
manure removal: slurry with perforated flooring	housing	38	-0.404	0.21
type of milking stalls: tandem type	milking	39	-0.404	0.19
provision of supplementary concentrate: manual, two times a day	feed	40	-0.390	0.37
cubicle housing system: deep bed cubicles and slatted floors	housing	41	-0.389	0.30
walkway floor in free-stall for dry cows: concrete slits	housing	42	-0.388	0.29
claw trimming frequency: three times per year	husbandry	43	-0.375	0.36
manure removal in free-stall for young stock: slits	housing	44	-0.375	0.31
manure removal: solid manure	housing	45	0.370	0.18
number of milking places	milking	46	-0.363	0.02
used forage types in diet: field forage silage, grass silage, hay, corn silage, pasture	feed	47	-0.362	0.36
provision of supplementary concentrate: electronic feeder	feed	48	0.349	0.34
type of milking stalls: herringbone parlour	milking	49	-0.332	0.36
floor in open-air area for lactating cows: no open-air areas	housing	50	-0.327	0.35
litter in free-stall for lactating cows: other	housing	51	0.326	0.28
manure removal in free-stall for lactating cows: slits	housing	52	-0.320	0.50
standard deviation content of crude fibre for dry cows	feed	53	0.320	0.39

Table 1. Cluster 1 z-score *(continued)*

Variable	Category	Z-Score-Rank	Z-Score	P-Value
dry cows group management: with lactating cows	husbandry	54	-0.317	0.36
free-stall system for dry cows: deep litter	housing	55	-0.315	0.60
claw trimming frequency: once per year	husbandry	56	-0.312	0.46
free-stall system for young stock: sloped floor	housing	57	-0.305	0.60
cubicle housing system: high bed cubicles and slatted floors	housing	58	-0.303	0.60
dry cows group management: separate	husbandry	59	0.302	0.37
floor in walkway of free-stall for young stock: solid concrete	housing	60	0.298	0.46
barn design: tie stall facility with pasture	housing	61	0.295	0.27
manure removal in free-stall for dry cows: scraper	housing	62	0.287	0.50
walkway floor in free-stall for lactating cows: rubberised slits	housing	63	-0.283	1.00
claw trimming frequency: only for lame animals	husbandry	64	-0.283	0.60
barn design: other	housing	65	-0.278	0.60
mean yearly precipitation	environment	66	0.272	0.37
forage type: partial mixed ration	feed	67	0.270	0.47
walkway floor in free-stall for dry cows: other	housing	68	-0.266	0.69
provision of supplementary concentrate: exact	feed	69	-0.266	1.00
milking take off: present	milking	70	0.266	0.34
litter in free-stall for lactating cows: long straw	housing	71	-0.263	0.69
floor in open-air area for young stock: solid concrete	housing	72	-0.261	0.51
forage type: total mixed ration	feed	73	-0.259	1.00
used forage types in diet: corn silage, hay, pasture	feed	74	-0.255	1.00
provision of concentrates: total mix ration	feed	75	-0.254	1.00
manure removal: slurry with solid flooring	housing	76	0.248	0.37
number of high wind days	environment	77	-0.243	0.23
standard deviation for dietary proportion of concentrates for dry cows	feed	78	0.224	0.37
forage type: grass and grass products plus corn only	feed	79	0.211	0.35
manure removal in free-stall for dry cows: other	housing	80	0.206	0.49
provision of concentrates: exact	feed	81	0.204	0.73
milking stimulation	milking	82	-0.194	0.53
mean content of crude fibre for lactating cows	feed	83	0.194	0.33
main breed: Brown Swiss	husbandry	84	0.192	0.50
forage type: year-round silage (with corn silage)	feed	85	-0.188	0.70
standard deviation for dietary proportion of concentrates for lactating cows	feed	86	-0.186	0.42
used forage types in diet: field forage silage, grass silage, hay, corn silage	feed	87	-0.180	1.00
used forage types in diet: grass silage, hay, pasture	feed	88	0.179	0.62
young stock are kept in a tie stall facility	housing	89	0.176	0.63
manure removal in free-stall for lactating cows: scraper	housing	90	0.172	0.74
feed quality problematic	feed	91	-0.172	0.51
lactating cows are kept in a tie stall facility	housing	92	0.163	0.63
manure removal in free-stall for lactating cows: other	housing	93	0.162	0.70
forage type: other	feed	94	-0.159	1.00
automated milking switch-off	milking	95	0.151	0.74
floor in walkway of free-stall for young stock walkway: other	housing	96	0.145	0.65
manure removal: mixed forms	housing	97	-0.145	1.00
used forage types in diet: other	feed	98	0.142	0.76
cubicle housing system: other	housing	99	0.141	0.64
manure removal in free-stall for young stock: other	housing	100	-0.136	0.74
herd size	husbandry	101	-0.135	0.66
main breed: Holstein	husbandry	102	-0.133	1.00
farm organically managed	husbandry	103	-0.121	1.00
walkway floor in free-stall for lactating cows: concrete slits	housing	104	-0.119	1.00
forage type: sequentially fed forages	feed	105	-0.115	0.74
floor in walkway of free-stall for young stock: solid concrete with slits	housing	106	0.113	0.54
forage type: mixed ration with concentrates	feed	107	0.111	0.74
milking vacuum	milking	108	0.104	0.61
provision of supplementary concentrate: other	feed	109	0.100	0.66
dry cows kept in tie stall facility	housing	110	0.098	0.66

Table 1. Cluster 1 z-score *(continued)*

Variable	Category	Z-Score-Rank	Z-Score	P-Value
type of milking stalls: pipe milking	milking	111	0.096	0.66
free-stall system for young stock: other	housing	112	0.096	0.67
free-stall system for young stock: deep litter	housing	113	0.096	0.67
annual herd milk yield average	husbandry	114	0.094	0.74
walkway floor in free-stall for dry cows: solid concrete with slits	housing	115	-0.088	1.00
main breed: Fleckvieh	husbandry	116	-0.079	0.77
standard deviation of yearly precipitation	environment	117	0.074	0.76
provision of concentrates: manual	feed	118	-0.072	1.00
litter in free-stall for lactating cows: chopped straw	housing	119	-0.071	1.00
mean yearly relative humidity	environment	120	-0.071	0.76
forage type: grass and grass products only	feed	121	0.065	0.73
forage type: grass plus corn and grass products plus corn	feed	122	-0.063	1.00
silo type: no silo	feed	123	-0.061	1.00
floor in open-air area for dry cows: no open-air areas	housing	124	0.059	1.00
walkway floor in free-stall for lactating cows: solid concrete	housing	125	-0.058	1.00
free-stall system for young stock: deep bed cubicle	housing	126	0.056	1.00
free-stall system for lactating cows: deep bed cubicle	housing	127	0.055	1.00
standard deviation of yearly relative humidity	environment	128	0.049	0.91
walkway floor in free-stall for dry cows: solid concrete	housing	129	0.043	1.00
free-stall system for dry cows: deep bed cubicle	housing	130	0.040	1.00
floor in open-air areas for dry cows: solid concrete	housing	131	-0.038	1.00
used forage types in diet: grass silage, hay	feed	132	0.037	1.00
floor in open-air areas for dry cows: other	housing	133	-0.036	1.00
dietary proportion of concentrates for dry cows	feed	134	-0.032	0.88
walkway floor in free-stall for lactating cows: other	housing	135	-0.027	1.00
floor in open air area for young stock: no open-air areas	housing	136	0.025	1.00
cubicle housing system: high bed cubicles and solid floors	housing	137	-0.024	1.00
dry cows group management: with young stock	husbandry	138	0.017	1.00
walkway floor in free-stall for lactating cows: solid concrete with slits	housing	139	0.016	1.00
free-stall system for young stock: high bed cubicle	housing	140	-0.014	1.00
floor in open-air areas for lactating cows: solid concrete	housing	141	0.013	1.00
dietary proportion of concentrates for lactating cows	feed	142	-0.010	0.96

Table 2. Cluster 2 z-score. Scores are ranked according to the absolute value, the highest value is assigned rank 1 and the lowest rank 142.

Variable	Category	Z-Score-Rank	Z-Score	P-Value
altitude	environment	1	1.14	0.00
young stock on alpine pasture	husbandry	2	1.07	0.00
mean content of crude fibre for lactating cows	feed	3	0.80	0.00
dry cows on alpine pasture	husbandry	4	0.76	0.00
mean yearly relative humidity	environment	5	-0.74	0.00
silo type: silage bales	feed	6	0.70	0.00
standard deviation of yearly temperature	environment	7	-0.67	0.00
forage type: grass and grass products plus corn only	feed	8	0.66	0.00
number of low temperature days	environment	9	0.65	0.00
floor in open air area for young stock: no open-air areas	housing	10	-0.63	0.00
number of high wind days	environment	11	-0.60	0.00
lactating cows are kept in a tie stall facility	housing	12	0.59	0.01
herd size	husbandry	13	-0.58	0.00
pasture of young stock	husbandry	14	0.57	0.00
barn design: other	housing	15	0.56	0.01
free-stall system for lactating cows: deep bed cubicle	housing	16	0.53	0.01
silo type: bunker silo	feed	17	-0.51	0.02
milking vacuum	milking	18	0.51	0.00
mean yearly temperature	environment	19	-0.50	0.00
floor in open-air area for young stock: solid concrete	housing	20	0.49	0.03
type of milking stalls: pipe milking	milking	21	0.49	0.03
young stock are kept in a tie stall facility	housing	22	0.48	0.02
number of milking places	milking	23	-0.47	0.00
free-stall system for young stock: high bed cubicle	housing	24	-0.47	0.06
mean content of crude fibre for dry cows	feed	25	0.46	0.03
free-stall system for young stock: deep bed cubicle	housing	26	0.45	0.08
milking unit removal: none	milking	27	0.44	0.02
free-stall system for dry cows: high bed cubicle	housing	28	-0.44	0.07
floor in open-air area for dry cows: no open-air areas	housing	29	-0.43	0.05
cubicle housing system: other	housing	30	0.43	0.04
manure removal: slurry with solid flooring	housing	31	-0.42	0.04
standard deviation of yearly precipitation	environment	32	-0.42	0.01
barn design: free-stall barn	housing	33	-0.41	0.04
free-stall system for lactating cows: high bed cubicle	housing	34	-0.41	0.13
manure removal: solid manure	housing	35	0.40	0.08
dietary proportion of concentrates for lactating cows	feed	36	-0.39	0.08
walkway floor in free-stall for lactating cows: rubberised slits	housing	37	0.38	0.12
floor in open-air areas for lactating cows: solid concrete	housing	38	0.38	0.07
manure removal: mixed forms	housing	39	0.38	0.09
claw trimming frequency: three times per year	husbandry	40	-0.38	0.08
free-stall system for dry cows: deep bed cubicle	housing	41	0.37	0.12
walkway floor in free-stall for dry cows: other	housing	42	0.35	0.20
walkway floor in free-stall for lactating cows: other	housing	43	0.35	0.20
manure removal in free-stall for lactating cows: scraper	housing	44	-0.33	0.19
floor in open-air area for lactating cows: no open-air areas	housing	45	-0.33	0.11
milking unit removal: present, including post-milking technology	milking	46	-0.33	0.13
main breed: Brown Swiss	husbandry	47	0.33	0.12
floor in open-air areas for dry cows: other	housing	48	0.32	0.13
lactating cows on alpine pasture	husbandry	49	0.32	0.11
barn design: tie stall facility with pasture	housing	50	0.32	0.11
floor in open-air area for young stock: unpaved	housing	51	0.32	0.15
walkway floor in free-stall for dry cows: solid concrete with slits	housing	52	0.31	0.24
used forage types in diet: grass silage, green forage, hay, pasture	feed	53	0.31	0.14
manure removal in free-stall for lactating cows: slits	housing	54	0.30	0.27
forage type: year-round silage (with corn silage)	feed	55	-0.30	0.21
free-stall system for lactating cows: other	housing	56	-0.30	0.36
forage type: sequentially fed forages	feed	57	0.29	0.15

Table 2. Cluster 2 z-score *(continued)*

Variable	Category	Z-Score-Rank	Z-Score	P-Value
used forage types in diet: field forage silage, grass silage, hay, corn silage	feed	58	-0.29	0.20
dry cows kept in tie stall facility	housing	59	0.28	0.18
provision of concentrates: manual	feed	60	0.27	0.16
milking take off: present	milking	61	-0.27	0.22
forage type: total mixed ration	feed	62	-0.26	0.36
walkway floor in free-stall for lactating cows: solid concrete	housing	63	-0.26	0.36
provision of concentrates: total mix ration	feed	64	-0.25	0.36
main breed: Holstein	husbandry	65	-0.25	0.31
feed quality problematic	feed	66	-0.25	0.09
provision of supplementary concentrate: manual, two times a day	feed	67	0.25	0.20
manure removal in free-stall for young stock: slits	housing	68	-0.25	0.40
floor in open-air areas for dry cows: solid concrete	housing	69	0.24	0.32
forage type: mixed ration with concentrates	feed	70	-0.24	0.32
number of high temperature days	environment	71	-0.24	0.17
used forage types in diet: grass silage, hay	feed	72	0.24	0.21
walkway floor in free-stall for lactating cows: rubber mats	housing	73	-0.24	0.47
milking stimulation	milking	74	-0.23	0.24
walkway floor in free-stall for dry cows: solid concrete	housing	75	-0.23	0.40
type of milking stalls: herringbone parlour	milking	76	-0.23	0.34
claw trimming frequency: once per year	husbandry	77	0.23	0.27
floor in walkway of free-stall for young stock: concrete slits	housing	78	-0.22	0.42
used forage types in diet: field forage silage, grass silage, hay, corn silage, pasture	feed	79	-0.22	0.47
walkway floor in free-stall for dry cows: concrete slits	housing	80	-0.22	0.40
farm organically managed	husbandry	81	0.21	0.28
cubicle housing system: deep bed cubicles and solid floors	housing	82	-0.21	0.37
manure removal in free-stall for dry cows: other	housing	83	0.21	0.39
manure removal in free-stall for young stock: other	housing	84	0.20	0.57
floor in walkway of free-stall for young stock: solid concrete	housing	85	0.20	0.39
manure removal in free-stall for dry cows: scraper	housing	86	-0.20	0.57
silo type: no silo	feed	87	-0.18	0.70
pasture of dry cows	husbandry	88	0.18	0.37
automated milking switch-off	milking	89	-0.18	0.42
type of milking stalls: side-by-side	milking	90	-0.17	0.70
standard deviation for dietary proportion of concentrates for lactating cows	feed	91	-0.17	0.39
forage type: partial mixed ration	feed	92	-0.17	0.45
cubicle housing system: high bed cubicles and slatted floors	housing	93	0.17	0.42
cubicle housing system: high bed cubicles and solid floors	housing	94	-0.16	0.70
walkway floor in free-stall for dry cows: rubber mats	housing	95	-0.16	0.69
provision of supplementary concentrate: other	feed	96	-0.13	0.74
provision of supplementary concentrate: electronic feeder	feed	97	-0.13	0.63
claw trimming frequency: twice per year	husbandry	98	0.13	0.65
dietary proportion of concentrates for dry cows	feed	99	-0.12	0.58
walkway floor in free-stall for lactating cows: solid concrete with slits	housing	100	-0.12	1.00
used forage types in diet: corn silage, hay, pasture	feed	101	0.12	0.63
standard deviation for dietary proportion of concentrates for dry cows	feed	102	-0.12	0.62
manure removal: slurry with perforated flooring	housing	103	-0.12	0.64
claw trimming frequency: only for lame animals	husbandry	104	-0.12	1.00
main breed: Fleckvieh	husbandry	105	-0.12	0.64
mean yearly precipitation	environment	106	-0.12	0.41
free-stall system for young stock: sloped floor	housing	107	0.11	0.64
free-stall system for dry cows: deep litter	housing	108	-0.11	1.00
provision of concentrates: exact	feed	109	-0.11	0.61
litter in free-stall for lactating cows: chopped straw	housing	110	0.10	0.80
forage type: grass and grass products only	feed	111	0.09	0.60
dry cows group management: with lactating cows	husbandry	112	-0.09	0.82
floor in walkway of free-stall for young stock walkway: other	housing	113	0.09	0.72

Table 2. Cluster 2 z-score *(continued)*

Variable	Category	Z-Score-Rank	Z-Score	P-Value
provision of supplementary concentrate: exact	feed	114	0.08	0.65
cubicle housing system: deep bed cubicles and slatted floors	housing	115	-0.08	0.80
floor in open-air areas for lactating cows: other	housing	116	-0.08	1.00
litter in free-stall for lactating cows: long straw	housing	117	-0.08	1.00
barn design: outdoor climate house closed	housing	118	-0.07	1.00
annual herd milk yield average	husbandry	119	-0.07	0.72
standard deviation content of crude fibre for lactating cows	feed	120	-0.07	0.74
used forage types in diet: grass silage, hay, pasture	feed	121	-0.06	1.00
dry cows group management: separate	husbandry	122	0.06	0.82
claw trimming done by farmer	husbandry	123	-0.06	0.80
manure removal in free-stall for young stock: scraper	housing	124	0.05	0.73
forage type: grass plus corn and grass products plus corn	feed	125	-0.05	1.00
used forage types in diet: other	feed	126	0.05	0.82
floor in walkway of free-stall for young stock: solid concrete with slits	housing	127	-0.05	1.00
litter in free-stall for lactating cows: other	housing	128	-0.05	1.00
standard deviation of yearly relative humidity	environment	129	-0.05	0.80
dry cows group management: with young stock	husbandry	130	0.04	0.69
manure removal in free-stall for lactating cows: other	housing	131	0.04	0.76
type of milking stalls: milking robot	milking	132	0.04	0.69
walkway floor in free-stall for lactating cows: concrete slits	housing	133	-0.04	1.00
free-stall system for young stock: other	housing	134	0.03	1.00
free-stall system for young stock: deep litter	housing	135	0.03	1.00
standard deviation content of crude fibre for dry cows	feed	136	-0.03	0.92
type of milking stalls: tandem type	milking	137	-0.03	1.00
pasture of lactating cows	husbandry	138	0.02	1.00
forage type: other	feed	139	-0.02	1.00
free-stall system for dry cows: other	housing	140	0.02	1.00
barn design: outdoor climate house open front	housing	141	0.00	1.00
manure removal in free-stall for dry cows: slits	housing	142	0.00	1.00

Table 3. Cluster 3 z-score. Scores are ranked according to the absolute value, the highest value is assigned rank 1 and the lowest rank 142.

Variable	Category	Z-Score-Rank	Z-Score	P-Value
number of high temperature days	environment	1	-0.50	0.00
mean yearly precipitation	environment	2	0.43	0.01
silo type: no silo	feed	3	0.39	0.01
free-stall system for dry cows: deep bed cubicle	housing	4	0.39	0.01
type of milking stalls: pipe milking	milking	5	0.38	0.01
manure removal in free-stall for lactating cows: scraper	housing	6	0.34	0.02
milking take off: present	milking	7	0.33	0.01
forage type: grass and grass products only	feed	8	0.32	0.02
pasture of lactating cows	husbandry	9	0.32	0.02
altitude	environment	10	0.30	0.00
free-stall system for dry cows: other	housing	11	-0.30	0.07
floor in walkway of free-stall for young stock walkway: other	housing	12	0.29	0.07
mean yearly temperature	environment	13	-0.28	0.01
standard deviation of yearly precipitation	environment	14	0.27	0.08
standard deviation of yearly temperature	environment	15	-0.27	0.03
lactating cows are kept in a tie stall facility	housing	16	0.27	0.05
free-stall system for lactating cows: deep bed cubicle	housing	17	0.27	0.07
dry cows kept in tie stall facility	housing	18	0.26	0.07
manure removal in free-stall for dry cows: scraper	housing	19	0.26	0.10
barn design: tie stall facility with pasture	housing	20	0.26	0.06
silo type: silage bales	feed	21	-0.25	0.08
milking unit removal: present, including post-milking technology	milking	22	-0.25	0.07
cubicle housing system: other	housing	23	0.24	0.06
cubicle housing system: high bed cubicles and solid floors	housing	24	-0.24	0.07
standard deviation content of crude fibre for dry cows	feed	25	-0.24	0.03
forage type: year-round silage (with corn silage)	feed	26	-0.23	0.09
number of milking places	milking	27	0.23	0.19
dietary proportion of concentrates for dry cows	feed	28	-0.23	0.04
used forage types in diet: grass silage, hay	feed	29	0.23	0.09
manure removal in free-stall for dry cows: other	housing	30	-0.23	0.14
manure removal: slurry with solid flooring	housing	31	0.22	0.10
floor in walkway of free-stall for young stock: concrete slits	housing	32	-0.22	0.12
free-stall system for young stock: other	housing	33	-0.22	0.13
standard deviation for dietary proportion of concentrates for dry cows	feed	34	-0.22	0.07
manure removal in free-stall for lactating cows: slits	housing	35	-0.22	0.16
free-stall system for dry cows: deep litter	housing	36	-0.22	0.19
used forage types in diet: grass silage, hay, pasture	feed	37	0.21	0.10
standard deviation for dietary proportion of concentrates for lactating cows	feed	38	0.21	0.10
cubicle housing system: deep bed cubicles and solid floors	housing	39	0.21	0.12
pasture of dry cows	husbandry	40	0.20	0.15
manure removal: slurry with perforated flooring	housing	41	-0.20	0.15
litter in free-stall for lactating cows: other	housing	42	-0.20	0.26
free-stall system for lactating cows: other	housing	43	-0.19	0.29
litter in free-stall for lactating cows: chopped straw	housing	44	0.19	0.24
walkway floor in free-stall for lactating cows: concrete slits	housing	45	-0.18	0.27
provision of supplementary concentrate: exact	feed	46	0.18	0.17
free-stall system for young stock: sloped floor	housing	47	0.18	0.30
walkway floor in free-stall for lactating cows: rubberised slits	housing	48	-0.18	0.46
cubicle housing system: deep bed cubicles and slatted floors	housing	49	-0.17	0.23
milking stimulation	milking	50	0.17	0.20
manure removal in free-stall for young stock: slits	housing	51	-0.17	0.25
free-stall system for lactating cows: high bed cubicle	housing	52	-0.17	0.41
barn design: outdoor climate house open front	housing	53	-0.17	0.25
used forage types in diet: corn silage, hay, pasture	feed	54	-0.16	0.29
floor in open-air area for young stock: unpaved	housing	55	0.16	0.30
standard deviation of yearly relative humidity	environment	56	0.16	0.25
claw trimming frequency: only for lame animals	husbandry	57	0.16	0.31

Table 3. Cluster 3 z-score *(continued)*

Variable	Category	Z-Score-Rank	Z-Score	P-Value
forage type: sequentially fed forages	feed	58	0.16	0.33
milking unit removal: none	milking	59	-0.15	0.28
manure removal in free-stall for lactating cows: other	housing	60	-0.15	0.37
floor in open-air area for dry cows: no open-air areas	housing	61	-0.15	0.29
walkway floor in free-stall for lactating cows: solid concrete	housing	62	0.15	0.36
type of milking stalls: milking robot	milking	63	-0.15	0.36
cubicle housing system: high bed cubicles and slatted floors	housing	64	-0.14	0.36
free-stall system for young stock: deep litter	housing	65	0.14	0.31
herd size	husbandry	66	-0.14	0.24
pasture of young stock	husbandry	67	0.14	0.35
forage type: mixed ration with concentrates	feed	68	-0.13	0.34
forage type: partial mixed ration	feed	69	-0.13	0.41
dry cows on alpine pasture	husbandry	70	-0.13	0.44
manure removal in free-stall for young stock: scraper	housing	71	0.13	0.47
dry cows group management: with young stock	husbandry	72	-0.13	0.52
claw trimming done by farmer	husbandry	73	0.13	0.44
floor in open-air areas for dry cows: other	housing	74	0.12	0.38
dry cows group management: with lactating cows	husbandry	75	0.12	0.38
floor in open-air areas for lactating cows: other	housing	76	0.12	0.41
milking vacuum	milking	77	0.12	0.31
walkway floor in free-stall for lactating cows: other	housing	78	0.11	0.48
claw trimming frequency: twice per year	husbandry	79	-0.11	0.47
claw trimming frequency: once per year	husbandry	80	0.11	0.39
barn design: other	housing	81	-0.11	0.52
provision of concentrates: exact	feed	82	0.11	0.54
used forage types in diet: field forage silage, grass silage, hay, corn silage	feed	83	-0.10	0.62
feed quality problematic	feed	84	-0.10	0.40
type of milking stalls: side-by-side	milking	85	-0.10	0.56
lactating cows on alpine pasture	husbandry	86	0.10	0.53
floor in open-air areas for lactating cows: solid concrete	housing	87	-0.10	0.48
claw trimming frequency: three times per year	husbandry	88	-0.09	0.59
floor in walkway of free-stall for young stock: solid concrete with slits	housing	89	-0.09	0.73
young stock are kept in a tie stall facility	housing	90	0.09	0.57
used forage types in diet: field forage silage, grass silage, hay, corn silage, pasture	feed	91	-0.09	0.59
walkway floor in free-stall for lactating cows: rubber mats	housing	92	0.08	0.59
provision of concentrates: manual	feed	93	-0.08	0.66
main breed: Fleckvieh	husbandry	94	0.08	0.59
floor in open-air areas for dry cows: solid concrete	housing	95	0.07	0.58
used forage types in diet: grass silage, green forage, hay, pasture	feed	96	-0.07	0.73
annual herd milk yield average	husbandry	97	-0.07	0.58
mean content of crude fibre for dry cows	feed	98	0.07	0.52
type of milking stalls: herringbone parlour	milking	99	-0.07	0.72
manure removal in free-stall for young stock: other	housing	100	0.07	0.70
forage type: total mixed ration	feed	101	-0.06	1.00
manure removal: mixed forms	housing	102	-0.06	0.80
provision of supplementary concentrate: manual, two times a day	feed	103	-0.06	0.80
young stock on alpine pasture	husbandry	104	0.06	0.72
dietary proportion of concentrates for lactating cows	feed	105	-0.06	0.64
provision of concentrates: total mix ration	feed	106	-0.06	1.00
floor in walkway of free-stall for young stock: solid concrete	housing	107	0.06	0.68
number of high wind days	environment	108	0.06	0.70
dry cows group management: separate	husbandry	109	-0.05	0.73
main breed: Brown Swiss	husbandry	110	-0.05	0.84
type of milking stalls: tandem type	milking	111	-0.05	0.84
walkway floor in free-stall for dry cows: solid concrete	housing	112	-0.05	0.83
provision of supplementary concentrate: electronic feeder	feed	113	-0.05	0.71
floor in open-air area for young stock: solid concrete	housing	114	-0.05	0.84

Table 3. Cluster 3 z-score *(continued)*

Variable	Category	Z-Score-Rank	Z-Score	P-Value
forage type: other	feed	115	0.05	0.80
walkway floor in free-stall for dry cows: other	housing	116	0.05	0.81
free-stall system for dry cows: high bed cubicle	housing	117	-0.05	1.00
barn design: free-stall barn	housing	118	0.05	0.73
forage type: grass and grass products plus corn only	feed	119	-0.04	1.00
main breed: Holstein	husbandry	120	-0.04	1.00
free-stall system for young stock: deep bed cubicle	housing	121	-0.04	1.00
mean yearly relative humidity	environment	122	0.04	0.77
manure removal in free-stall for dry cows: slits	housing	123	-0.04	0.84
floor in open air area for young stock: no open-air areas	housing	124	-0.04	0.85
walkway floor in free-stall for dry cows: solid concrete with slits	housing	125	0.04	0.78
walkway floor in free-stall for dry cows: concrete slits	housing	126	-0.04	1.00
walkway floor in free-stall for lactating cows: solid concrete with slits	housing	127	-0.03	1.00
forage type: grass plus corn and grass products plus corn	feed	128	0.03	0.78
mean content of crude fibre for lactating cows	feed	129	0.03	0.82
number of low temperature days	environment	130	-0.03	0.83
standard deviation content of crude fibre for lactating cows	feed	131	0.02	0.86
silo type: bunker silo	feed	132	-0.02	1.00
manure removal: solid manure	housing	133	0.02	1.00
walkway floor in free-stall for dry cows: rubber mats	housing	134	0.02	1.00
automated milking switch-off	milking	135	0.02	1.00
floor in open-air area for lactating cows: no open-air areas	housing	136	0.02	1.00
litter in free-stall for lactating cows: long straw	housing	137	-0.02	1.00
used forage types in diet: other	feed	138	-0.01	1.00
farm organically managed	husbandry	139	0.01	1.00
barn design: outdoor climate house closed	housing	140	-0.01	1.00
free-stall system for young stock: high bed cubicle	housing	141	0.00	1.00
provision of supplementary concentrate: other	feed	142	0.00	1.00

Table 4. Cluster 4 z-score. Scores are ranked according to the absolute value, the highest value is assigned rank 1 and the lowest rank 142.

Variable	Category	Z-Score-Rank	Z-Score	P-Value
altitude	environment	1	-1.33	0.00
number of high temperature days	environment	2	1.11	0.00
standard deviation of yearly temperature	environment	3	1.03	0.00
mean yearly temperature	environment	4	0.92	0.00
young stock on alpine pasture	husbandry	5	-0.73	0.00
pasture of lactating cows	husbandry	6	-0.71	0.00
number of high wind days	environment	7	0.69	0.00
mean yearly precipitation	environment	8	-0.68	0.00
pasture of young stock	husbandry	9	-0.68	0.00
pasture of dry cows	husbandry	10	-0.66	0.00
mean content of crude fibre for lactating cows	feed	11	-0.65	0.00
forage type: year-round silage (with corn silage)	feed	12	0.64	0.00
forage type: grass and grass products only	feed	13	-0.53	0.00
used forage types in diet: field forage silage, grass silage, hay, corn silage	feed	14	0.49	0.01
number of low temperature days	environment	15	-0.49	0.00
silo type: bunker silo	feed	16	0.47	0.01
standard deviation of yearly relative humidity	environment	17	-0.45	0.00
floor in walkway of free-stall for young stock: concrete slits	housing	18	0.42	0.01
annual herd milk yield average	husbandry	19	0.40	0.03
type of milking stalls: pipe milking	milking	20	-0.39	0.01
dry cows on alpine pasture	husbandry	21	-0.39	0.02
free-stall system for young stock: high bed cubicle	housing	22	0.38	0.05
free-stall system for lactating cows: deep bed cubicle	housing	23	-0.37	0.04
milking unit removal: present, including post-milking technology	milking	24	0.37	0.04
manure removal in free-stall for young stock: slits	housing	25	0.36	0.03
dietary proportion of concentrates for lactating cows	feed	26	0.36	0.01
standard deviation of yearly precipitation	environment	27	-0.36	0.01
lactating cows are kept in a tie stall facility	housing	28	-0.36	0.05
barn design: outdoor climate house open front	housing	29	0.36	0.06
claw trimming frequency: three times per year	husbandry	30	0.35	0.06
herd size	husbandry	31	0.35	0.07
young stock are kept in a tie stall facility	housing	32	-0.35	0.05
used forage types in diet: grass silage, hay, pasture	feed	33	-0.35	0.04
forage type: total mixed ration	feed	34	0.35	0.07
walkway floor in free-stall for dry cows: rubber mats	housing	35	-0.35	0.07
used forage types in diet: corn silage, hay, pasture	feed	36	0.34	0.07
silo type: no silo	feed	37	-0.34	0.08
provision of concentrates: total mix ration	feed	38	0.32	0.08
floor in open air area for young stock: no open-air areas	housing	39	0.32	0.08
walkway floor in free-stall for lactating cows: rubber mats	housing	40	-0.31	0.08
lactating cows on alpine pasture	husbandry	41	-0.30	0.13
barn design: tie stall facility with pasture	housing	42	-0.30	0.13
silo type: silage bales	feed	43	-0.29	0.13
free-stall system for lactating cows: high bed cubicle	housing	44	0.28	0.13
claw trimming frequency: only for lame animals	husbandry	45	-0.28	0.13
floor in open-air area for young stock: unpaved	housing	46	-0.28	0.22
barn design: other	housing	47	-0.28	0.13
floor in open-air area for dry cows: no open-air areas	housing	48	0.28	0.10
farm organically managed	husbandry	49	-0.27	0.14
walkway floor in free-stall for dry cows: solid concrete with slits	housing	50	-0.27	0.20
milking take off: present	milking	51	-0.27	0.12
floor in walkway of free-stall for young stock walkway: other	housing	52	-0.26	0.16
mean yearly relative humidity	environment	53	0.26	0.01
used forage types in diet: grass silage, green forage, hay, pasture	feed	54	-0.25	0.21
manure removal in free-stall for young stock: other	housing	55	-0.25	0.14
manure removal: slurry with perforated flooring	housing	56	0.25	0.14
free-stall system for young stock: deep litter	housing	57	-0.24	0.25

Table 4. Cluster 4 z-score *(continued)*

Variable	Category	Z-Score-Rank	Z-Score	P-Value
free-stall system for dry cows: deep bed cubicle	housing	58	-0.24	0.19
main breed: Holstein	husbandry	59	0.24	0.22
standard deviation for dietary proportion of concentrates for lactating cows	feed	60	0.23	0.30
walkway floor in free-stall for dry cows: solid concrete	housing	61	0.23	0.23
number of milking places	milking	62	0.23	0.28
provision of supplementary concentrate: other	feed	63	0.22	0.23
floor in open-air areas for dry cows: solid concrete	housing	64	-0.22	0.21
floor in walkway of free-stall for young stock: solid concrete	housing	65	-0.22	0.26
cubicle housing system: deep bed cubicles and solid floors	housing	66	-0.21	0.22
walkway floor in free-stall for lactating cows: concrete slits	housing	67	0.21	0.23
free-stall system for lactating cows: other	housing	68	0.21	0.25
floor in open-air areas for lactating cows: solid concrete	housing	69	-0.21	0.30
walkway floor in free-stall for lactating cows: solid concrete	housing	70	0.21	0.22
cubicle housing system: high bed cubicles and slatted floors	housing	71	0.19	0.27
manure removal in free-stall for dry cows: scraper	housing	72	-0.19	0.37
manure removal: mixed forms	housing	73	-0.19	0.37
provision of supplementary concentrate: manual, two times a day	feed	74	-0.19	0.37
free-stall system for young stock: deep bed cubicle	housing	75	-0.18	0.41
free-stall system for young stock: sloped floor	housing	76	-0.17	0.46
floor in open-air area for young stock: solid concrete	housing	77	-0.17	0.36
free-stall system for dry cows: other	housing	78	0.17	0.39
cubicle housing system: high bed cubicles and solid floors	housing	79	0.17	0.30
floor in open-air area for lactating cows: no open-air areas	housing	80	0.16	0.41
litter in free-stall for lactating cows: other	housing	81	-0.16	0.46
walkway floor in free-stall for dry cows: concrete slits	housing	82	0.16	0.46
cubicle housing system: other	housing	83	-0.16	0.53
dry cows group management: with lactating cows	husbandry	84	0.16	0.41
used forage types in diet: grass silage, hay	feed	85	-0.14	0.69
provision of concentrates: manual	feed	86	-0.14	0.61
forage type: partial mixed ration	feed	87	-0.14	0.47
feed quality problematic	feed	88	0.14	0.42
manure removal in free-stall for lactating cows: scraper	housing	89	-0.14	0.53
used forage types in diet: field forage silage, grass silage, hay, corn silage, pasture	feed	90	-0.14	0.53
manure removal in free-stall for young stock: scraper	housing	91	-0.14	0.60
standard deviation content of crude fibre for dry cows	feed	92	0.13	0.45
dry cows group management: separate	husbandry	93	-0.13	0.54
cubicle housing system: deep bed cubicles and slatted floors	housing	94	0.13	0.48
litter in free-stall for lactating cows: long straw	housing	95	0.13	0.44
provision of supplementary concentrate: exact	feed	96	-0.13	0.69
manure removal in free-stall for lactating cows: slits	housing	97	0.13	0.52
type of milking stalls: side-by-side	milking	98	0.12	0.50
forage type: grass plus corn and grass products plus corn	feed	99	0.12	0.50
milking stimulation	milking	100	-0.11	0.52
type of milking stalls: tandem type	milking	101	0.11	0.49
forage type: grass and grass products plus corn only	feed	102	-0.11	0.74
floor in open-air areas for dry cows: other	housing	103	-0.11	0.74
manure removal in free-stall for dry cows: slits	housing	104	0.11	0.65
main breed: Brown Swiss	husbandry	105	-0.10	0.64
claw trimming frequency: once per year	husbandry	106	-0.09	0.80
walkway floor in free-stall for lactating cows: other	housing	107	-0.09	0.80
walkway floor in free-stall for lactating cows: solid concrete with slits	housing	108	-0.09	0.74
manure removal in free-stall for dry cows: other	housing	109	0.09	0.64
dry cows kept in tie stall facility	housing	110	-0.09	0.77
free-stall system for dry cows: high bed cubicle	housing	111	0.09	0.57
free-stall system for dry cows: deep litter	housing	112	0.08	0.71
standard deviation content of crude fibre for lactating cows	feed	113	0.08	0.66
main breed: Fleckvieh	husbandry	114	-0.07	0.68

Table 4. Cluster 4 z-score *(continued)*

Variable	Category	Z-Score-Rank	Z-Score	P-Value
manure removal: slurry with solid flooring	housing	115	-0.07	0.83
claw trimming done by farmer	husbandry	116	-0.07	0.66
type of milking stalls: herringbone parlour	milking	117	0.07	0.68
manure removal: solid manure	housing	118	-0.07	1.00
floor in open-air areas for lactating cows: other	housing	119	0.07	0.75
dietary proportion of concentrates for dry cows	feed	120	-0.06	0.73
free-stall system for young stock: other	housing	121	0.06	0.77
walkway floor in free-stall for dry cows: other	housing	122	0.05	0.79
barn design: free-stall barn	housing	123	0.05	0.84
milking vacuum	milking	124	0.05	0.69
forage type: sequentially fed forages	feed	125	-0.05	0.82
type of milking stalls: milking robot	milking	126	0.05	0.72
provision of concentrates: exact	feed	127	-0.05	0.82
provision of supplementary concentrate: electronic feeder	feed	128	0.04	1.00
floor in walkway of free-stall for young stock: solid concrete with slits	housing	129	-0.04	1.00
litter in free-stall for lactating cows: chopped straw	housing	130	0.04	0.83
dry cows group management: with young stock	husbandry	131	-0.04	1.00
automated milking switch-off	milking	132	-0.03	1.00
milking unit removal: none	milking	133	0.03	1.00
manure removal in free-stall for lactating cows: other	housing	134	0.02	1.00
walkway floor in free-stall for lactating cows: rubberised slits	housing	135	-0.02	1.00
mean content of crude fibre for dry cows	feed	136	0.02	0.91
forage type: mixed ration with concentrates	feed	137	-0.02	1.00
barn design: outdoor climate house closed	housing	138	-0.01	1.00
claw trimming frequency: twice per year	husbandry	139	-0.01	1.00
standard deviation for dietary proportion of concentrates for dry cows	feed	140	0.00	0.98
forage type: other	feed	141	0.00	1.00
used forage types in diet: other	feed	142	0.00	1.00

Table 5. Cluster 5 z-score. Scores are ranked according to the absolute value, the highest value is assigned rank 1 and the lowest rank 142.

Variable	Category	Z-Score-Rank	Z-Score	P-Value
altitude	environment	1	-0.49	0.00
number of low temperature days	environment	2	-0.44	0.00
mean yearly temperature	environment	3	0.43	0.00
milking vacuum	milking	4	-0.35	0.01
number of high temperature days	environment	5	0.34	0.00
mean content of crude fibre for dry cows	feed	6	-0.33	0.00
cubicle housing system: other	housing	7	-0.32	0.00
used forage types in diet: field forage silage, grass silage, hay, corn silage, pasture	feed	8	0.30	0.01
lactating cows are kept in a tie stall facility	housing	9	-0.30	0.00
young stock on alpine pasture	husbandry	10	-0.30	0.01
dry cows kept in tie stall facility	housing	11	-0.29	0.01
dry cows on alpine pasture	husbandry	12	-0.29	0.01
type of milking stalls: pipe milking	milking	13	-0.28	0.01
dietary proportion of concentrates for dry cows	feed	14	0.26	0.02
free-stall system for dry cows: deep bed cubicle	housing	15	-0.25	0.02
barn design: tie stall facility with pasture	housing	16	-0.24	0.04
manure removal: solid manure	housing	17	-0.22	0.05
forage type: grass and grass products plus corn only	feed	18	-0.22	0.06
standard deviation of yearly temperature	environment	19	0.22	0.02
used forage types in diet: grass silage, hay	feed	20	-0.21	0.06
free-stall system for dry cows: high bed cubicle	housing	21	0.20	0.06
floor in open-air area for young stock: unpaved	housing	22	-0.20	0.10
floor in open-air areas for lactating cows: other	housing	23	-0.20	0.08
forage type: sequentially fed forages	feed	24	-0.19	0.10
free-stall system for dry cows: deep litter	housing	25	0.19	0.13
forage type: mixed ration with concentrates	feed	26	0.19	0.10
herd size	husbandry	27	0.19	0.07
forage type: partial mixed ration	feed	28	0.19	0.12
cubicle housing system: deep bed cubicles and slatted floors	housing	29	0.18	0.09
lactating cows on alpine pasture	husbandry	30	-0.18	0.14
standard deviation for dietary proportion of concentrates for lactating cows	feed	31	-0.18	0.07
type of milking stalls: herringbone parlour	milking	32	0.17	0.13
cubicle housing system: high bed cubicles and solid floors	housing	33	0.17	0.15
litter in free-stall for lactating cows: other	housing	34	0.17	0.16
litter in free-stall for lactating cows: chopped straw	housing	35	-0.17	0.16
standard deviation for dietary proportion of concentrates for dry cows	feed	36	0.17	0.13
manure removal: slurry with perforated flooring	housing	37	0.16	0.17
barn design: outdoor climate house closed	housing	38	0.16	0.18
free-stall system for lactating cows: high bed cubicle	housing	39	0.16	0.15
floor in open-air areas for dry cows: other	housing	40	-0.15	0.27
mean yearly relative humidity	environment	41	0.15	0.14
free-stall system for lactating cows: deep bed cubicle	housing	42	-0.15	0.16
feed quality problematic	feed	43	0.14	0.22
manure removal in free-stall for young stock: scraper	housing	44	-0.14	0.27
annual herd milk yield average	husbandry	45	-0.14	0.20
claw trimming frequency: only for lame animals	husbandry	46	0.14	0.22
claw trimming done by farmer	husbandry	47	-0.13	0.27
claw trimming frequency: three times per year	husbandry	48	0.13	0.31
walkway floor in free-stall for dry cows: other	housing	49	-0.13	0.29
provision of supplementary concentrate: manual, two times a day	feed	50	0.13	0.24
silo type: bunker silo	feed	51	0.12	0.29
walkway floor in free-stall for lactating cows: other	housing	52	-0.12	0.30
standard deviation of yearly precipitation	environment	53	0.12	0.23
young stock are kept in a tie stall facility	housing	54	-0.12	0.30
floor in open-air area for dry cows: no open-air areas	housing	55	0.12	0.32
floor in walkway of free-stall for young stock walkway: other	housing	56	-0.11	0.47

Table 5. Cluster 5 z-score *(continued)*

Variable	Category	Z-Score-Rank	Z-Score	P-Value
standard deviation of yearly relative humidity	environment	57	0.11	0.26
silo type: silage bales	feed	58	-0.11	0.33
walkway floor in free-stall for lactating cows: solid concrete	housing	59	-0.11	0.32
claw trimming frequency: twice per year	husbandry	60	-0.11	0.40
free-stall system for young stock: other	housing	61	0.11	0.35
number of high wind days	environment	62	-0.10	0.31
walkway floor in free-stall for lactating cows: rubber mats	housing	63	0.10	0.34
floor in open air area for young stock: no open-air areas	housing	64	0.10	0.47
walkway floor in free-stall for lactating cows: solid concrete with slits	housing	65	0.10	0.39
dry cows group management: with young stock	husbandry	66	0.10	0.37
pasture of young stock	husbandry	67	-0.09	0.38
floor in open-air area for lactating cows: no open-air areas	housing	68	0.09	0.41
walkway floor in free-stall for dry cows: concrete slits	housing	69	0.09	0.44
floor in walkway of free-stall for young stock: solid concrete with slits	housing	70	0.09	0.52
main breed: Brown Swiss	husbandry	71	-0.08	0.57
manure removal in free-stall for lactating cows: scraper	housing	72	-0.08	0.49
provision of supplementary concentrate: other	feed	73	-0.08	0.63
type of milking stalls: tandem type	milking	74	0.08	0.57
dry cows group management: with lactating cows	husbandry	75	-0.08	0.51
barn design: free-stall barn	housing	76	-0.07	0.51
manure removal in free-stall for dry cows: scraper	housing	77	-0.07	0.59
milking take off: present	milking	78	-0.07	0.59
farm organically managed	husbandry	79	0.07	0.55
floor in walkway of free-stall for young stock: concrete slits	housing	80	0.07	0.59
standard deviation content of crude fibre for lactating cows	feed	81	0.07	0.53
manure removal in free-stall for dry cows: slits	housing	82	0.06	0.59
manure removal in free-stall for young stock: other	housing	83	0.06	0.59
claw trimming frequency: once per year	husbandry	84	-0.06	0.68
cubicle housing system: deep bed cubicles and solid floors	housing	85	-0.06	0.62
standard deviation content of crude fibre for dry cows	feed	86	0.06	0.57
barn design: other	housing	87	0.06	0.75
mean content of crude fibre for lactating cows	feed	88	-0.06	0.58
milking stimulation	milking	89	0.06	0.73
provision of concentrates: exact	feed	90	-0.06	0.71
walkway floor in free-stall for lactating cows: rubberised slits	housing	91	0.05	0.75
provision of supplementary concentrate: exact	feed	92	-0.05	0.75
type of milking stalls: milking robot	milking	93	-0.05	0.77
manure removal in free-stall for young stock: slits	housing	94	0.05	0.72
forage type: grass plus corn and grass products plus corn	feed	95	-0.05	0.79
barn design: outdoor climate house open front	housing	96	0.05	0.67
pasture of dry cows	husbandry	97	-0.05	0.74
milking unit removal: none	milking	98	0.05	0.74
manure removal in free-stall for lactating cows: slits	housing	99	0.04	0.72
manure removal in free-stall for lactating cows: other	housing	100	0.04	0.69
main breed: Holstein	husbandry	101	0.04	0.81
main breed: Fleckvieh	husbandry	102	0.04	0.74
free-stall system for young stock: deep bed cubicle	housing	103	-0.04	0.82
free-stall system for young stock: high bed cubicle	housing	104	-0.04	0.86
provision of concentrates: manual	feed	105	0.04	0.84
forage type: total mixed ration	feed	106	0.04	0.74
provision of concentrates: total mix ration	feed	107	0.04	0.74
automated milking switch-off	milking	108	0.04	0.85
used forage types in diet: other	feed	109	-0.04	0.74
walkway floor in free-stall for lactating cows: concrete slits	housing	110	0.04	0.85
silo type: no silo	feed	111	-0.04	1.00
floor in open-air areas for lactating cows: solid concrete	housing	112	0.03	0.87
used forage types in diet: grass silage, green forage, hay, pasture	feed	113	-0.03	1.00
used forage types in diet: corn silage, hay, pasture	feed	114	-0.03	1.00
floor in walkway of free-stall for young stock: solid concrete	housing	115	-0.03	0.85

Table 5. Cluster 5 z-score *(continued)*

Variable	Category	Z-Score-Rank	Z-Score	P-Value
walkway floor in free-stall for dry cows: rubber mats	housing	116	0.03	0.78
type of milking stalls: side-by-side	milking	117	-0.03	1.00
milking unit removal: present, including post-milking technology	milking	118	0.03	0.79
free-stall system for lactating cows: other	housing	119	0.03	0.77
forage type: grass and grass products only	feed	120	-0.03	0.85
floor in open-air areas for dry cows: solid concrete	housing	121	-0.02	0.86
number of milking places	milking	122	-0.02	0.79
walkway floor in free-stall for dry cows: solid concrete with slits	housing	123	0.02	1.00
manure removal: mixed forms	housing	124	0.02	0.81
dietary proportion of concentrates for lactating cows	feed	125	0.02	0.83
manure removal: slurry with solid flooring	housing	126	-0.02	0.87
dry cows group management: separate	husbandry	127	0.02	0.87
litter in free-stall for lactating cows: long straw	housing	128	0.02	0.83
forage type: year-round silage (with corn silage)	feed	129	0.02	0.82
free-stall system for young stock: sloped floor	housing	130	-0.01	1.00
walkway floor in free-stall for dry cows: solid concrete	housing	131	-0.01	1.00
pasture of lactating cows	husbandry	132	0.01	1.00
used forage types in diet: field forage silage, grass silage, hay, corn silage	feed	133	-0.01	1.00
cubicle housing system: high bed cubicles and slatted floors	housing	134	0.01	1.00
free-stall system for dry cows: other	housing	135	-0.01	1.00
used forage types in diet: grass silage, hay, pasture	feed	136	-0.01	1.00
manure removal in free-stall for dry cows: other	housing	137	0.01	1.00
floor in open-air area for young stock: solid concrete	housing	138	0.01	1.00
forage type: other	feed	139	0.01	1.00
provision of supplementary concentrate: electronic feeder	feed	140	0.00	1.00
free-stall system for young stock: deep litter	housing	141	0.00	1.00
mean yearly precipitation	environment	142	0.00	0.99

Table 6. Descriptive statistics for each cluster. For numeric variables the mean and standard deviation is reported, while for binary variables the percentage. Variables are listed in alphabetical order.

Variable	Statistic	Cluster 1	Cluster 2	Cluster 3	Cluster 4	Cluster 5	Unit
altitude	Mean (SD)	1169.75 ±134.52	900 ±0	695.47 ±29.6	301.38 ±14.32	504.26 ±31.49	m
annual herd milk yield average	Mean (SD)	8761.24 ±1459.86	8529.82 ±1343.6	8524.95 ±1368.86	9200.95 ±1501.51	8421.15 ±1504.74	kg
automated milking switch-off	Percentage	83.33	69.57	77.78	75.86	77.19	
barn design: free-stall barn	Percentage	83.33	21.74	44.44	44.83	38.6	
barn design: other	Percentage	0	21.74	4.44	0	8.77	
barn design: outdoor climate house closed	Percentage	0	21.74	24.44	24.14	31.58	
barn design: outdoor climate house open front	Percentage	0	17.39	11.11	31.03	19.3	
barn design: tie stall facility with pasture	Percentage	16.67	17.39	15.56	0	1.75	
claw trimming done by farmer	Percentage	91.67	69.57	77.78	68.97	64.91	
claw trimming frequency: once per year	Percentage	8.33	30.43	24.44	17.24	17.54	
claw trimming frequency: only for lame animals	Percentage	0	4.35	11.11	0	10.53	
claw trimming frequency: three times per year	Percentage	0	0	8.89	24.14	15.79	
claw trimming frequency: twice per year	Percentage	91.67	65.22	51.11	58.62	50.88	
cubicle housing system: deep bed cubicles and slatted floors	Percentage	8.33	21.74	17.78	31.03	33.33	
cubicle housing system: deep bed cubicles and solid floors	Percentage	66.67	34.78	55.56	34.48	42.11	
cubicle housing system: high bed cubicles and slatted floors	Percentage	0	13.04	4.44	13.79	8.77	
cubicle housing system: high bed cubicles and solid floors	Percentage	8.33	4.35	2.22	13.79	14.04	
cubicle housing system: other	Percentage	16.67	26.09	20	6.9	1.75	
dietary proportion of concentrates for dry cows	Mean (SD)	0.08 ±0.05	0.07 ±0.08	0.07 ±0.05	0.08 ±0.07	0.1 ±0.08	
dietary proportion of concentrates for lactating cows	Mean (SD)	0.32 ±0.05	0.29 ±0.09	0.32 ±0.09	0.35 ±0.06	0.32 ±0.08	
dry cows group management: separate	Percentage	66.67	52.17	48.89	44.83	52.63	
dry cows group management: with lactating cows	Percentage	25	34.78	46.67	48.28	36.84	
dry cows group management: with young stock	Percentage	8.33	8.7	4.44	6.9	10.53	
dry cows kept in tie stall facility	Percentage	16.67	21.74	22.22	10.34	3.51	
dry cows on alpine pasture	Percentage	58.33	39.13	8.89	0	3.51	
farm organically managed	Percentage	16.67	30.43	22.22	10.34	24.56	
feed quality problematic	Mean (SD)	0.17 ±0.23	0.15 ±0.17	0.19 ±0.22	0.25 ±0.26	0.25 ±0.3	
floor in open air area for young stock: no open-air areas	Percentage	58.33	26.09	57.78	72.41	57.89	
floor in open-air area for dry cows: no open-air areas	Percentage	50	26.09	42.22	65.52	54.39	
floor in open-air area for lactating cows: no open-air areas	Percentage	25	26.09	44.44	51.72	47.37	
floor in open-air area for young stock: solid concrete	Percentage	16.67	43.48	26.67	20.69	26.32	
floor in open-air area for young stock: unpaved	Percentage	16.67	13.04	11.11	0	1.75	
floor in open-air areas for dry cows: other	Percentage	8.33	17.39	13.33	6.9	5.26	
floor in open-air areas for dry cows: solid concrete	Percentage	33.33	43.48	40	27.59	35.09	
floor in open-air areas for lactating cows: other	Percentage	25	8.7	15.56	13.79	5.26	
floor in open-air areas for lactating cows: solid concrete	Percentage	41.67	60.87	40	34.48	45.61	
floor in walkway of free-stall for young stock walkway: other	Percentage	16.67	13.04	22.22	6.9	10.53	

Table 6. Descriptive statistics for each cluster.*(continued)*

Variable	Statistic	Cluster 1	Cluster 2	Cluster 3	Cluster 4	Cluster 5	Unit
floor in walkway of free-stall for young stock: concrete slits	Percentage	16.67	21.74	26.67	65.52	40.35	
floor in walkway of free-stall for young stock: solid concrete	Percentage	33.33	26.09	26.67	20.69	24.56	
floor in walkway of free-stall for young stock: solid concrete with slits	Percentage	8.33	4.35	4.44	6.9	8.77	
forage type: grass and grass products only	Percentage	25	26.09	35.56	0	21.05	
forage type: grass and grass products plus corn only	Percentage	16.67	30.43	8.89	6.9	3.51	
forage type: grass plus corn and grass products plus corn	Percentage	8.33	8.7	11.11	13.79	8.77	
forage type: mixed ration with concentrates	Percentage	33.33	17.39	22.22	27.59	36.84	
forage type: other	Percentage	8.33	13.04	15.56	13.79	14.04	
forage type: partial mixed ration	Percentage	33.33	17.39	17.78	17.24	31.58	
forage type: sequentially fed forages	Percentage	58.33	82.61	71.11	62.07	57.89	
forage type: total mixed ration	Percentage	0	0	4.44	13.79	7.02	
forage type: year-round silage (with corn silage)	Percentage	8.33	4.35	6.67	37.93	15.79	
free-stall system for dry cows: deep bed cubicle	Percentage	50	56.52	60	41.38	43.86	
free-stall system for dry cows: deep litter	Percentage	0	4.35	2.22	10.34	14.04	
free-stall system for dry cows: high bed cubicle	Percentage	0	0	11.11	17.24	22.81	
free-stall system for dry cows: other	Percentage	33.33	13.04	4.44	20.69	15.79	
free-stall system for lactating cows: deep bed cubicle	Percentage	66.67	73.91	71.11	62.07	70.18	
free-stall system for lactating cows: high bed cubicle	Percentage	0	0	6.67	24.14	19.3	
free-stall system for lactating cows: other	Percentage	16.67	0	2.22	13.79	8.77	
free-stall system for young stock: deep bed cubicle	Percentage	16.67	26.09	13.33	10.34	14.04	
free-stall system for young stock: deep litter	Percentage	16.67	13.04	17.78	6.9	14.04	
free-stall system for young stock: high bed cubicle	Percentage	33.33	13.04	33.33	55.17	33.33	
free-stall system for young stock: other	Percentage	16.67	13.04	6.67	17.24	17.54	
free-stall system for young stock: sloped floor	Percentage	0	8.7	11.11	3.45	7.02	
herd size	Mean (SD)	29.91 ±18.36	22.36 ±14.94	29.88 ±14.42	38.26 ±20.05	35.44 ±16.42	cows
lactating cows are kept in a tie stall facility	Percentage	16.67	30.43	20	0	1.75	
lactating cows on alpine pasture	Percentage	25	17.39	11.11	0	3.51	
litter in free-stall for lactating cows: chopped straw	Percentage	41.67	43.48	48.89	51.72	40.35	
litter in free-stall for lactating cows: long straw	Percentage	8.33	13.04	15.56	24.14	19.3	
litter in free-stall for lactating cows: other	Percentage	33.33	17.39	13.33	17.24	29.82	
main breed: Brown Swiss	Percentage	33.33	39.13	22.22	20.69	21.05	
main breed: Fleckvieh	Percentage	58.33	56.52	64.44	58.62	63.16	
main breed: Holstein	Percentage	8.33	4.35	11.11	20.69	14.04	
manure removal in free-stall for young stock: scraper	Percentage	33.33	13.04	20	13.79	12.28	
manure removal in free-stall for dry cows: other	Percentage	33.33	26.09	15.56	31.03	29.82	
manure removal in free-stall for dry cows: scraper	Percentage	41.67	17.39	37.78	24.14	31.58	
manure removal in free-stall for dry cows: slits	Percentage	8.33	21.74	24.44	34.48	35.09	
manure removal in free-stall for lactating cows: other	Percentage	25	17.39	13.33	24.14	24.56	
manure removal in free-stall for lactating cows: scraper	Percentage	41.67	17.39	46.67	34.48	36.84	
manure removal in free-stall for lactating cows: slits	Percentage	16.67	34.78	20	41.38	36.84	

Table 6. Descriptive statistics for each cluster.*(continued)*

Variable	Statistic	Cluster 1	Cluster 2	Cluster 3	Cluster 4	Cluster 5	Unit
manure removal in free-stall for young stock: other	Percentage	25	30.43	35.56	27.59	38.6	
manure removal in free-stall for young stock: slits	Percentage	16.67	17.39	26.67	58.62	38.6	
manure removal: mixed forms	Percentage	8.33	26.09	11.11	6.9	14.04	
manure removal: slurry with perforated flooring	Percentage	16.67	30.43	26.67	48.28	43.86	
manure removal: slurry with solid flooring	Percentage	50	17.39	48.89	34.48	36.84	
manure removal: solid manure	Percentage	25	26.09	13.33	10.34	5.26	
mean content of crude fibre for dry cows	Mean (SD)	256.17 ±30.04	256.1 ±23.41	246.51 ±17.95	245.35 ±29.15	236.63 ±24.28	
mean content of crude fibre for lactating cows	Mean (SD)	195.56 ±13.41	208.1 ±22.31	192.12 ±17.39	178.35 ±19.4	190.41 ±19.38	
mean yearly precipitation	Mean (SD)	3.03 ±0.73	2.76 ±0.45	3.13 ±0.92	2.37 ±0.23	2.84 ±0.56	mm
mean yearly relative humidity	Mean (SD)	80.4 ±2.47	78.37 ±3.61	80.74 ±3.52	81.39 ±1.08	81.08 ±2.8	%
mean yearly temperature	Mean (SD)	7.57 ±1.2	9.62 ±0.8	9.87 ±0.72	11.26 ±0.26	10.69 ±0.65	degree
milking stimulation	Percentage	58.33	56.52	75.56	62.07	70.18	
milking take off: present	Percentage	41.67	17.39	44.44	17.24	26.32	
milking unit removal: none	Percentage	33.33	82.61	53.33	62.07	63.16	
milking unit removal: present, including post-milking technology	Percentage	25	0	2.22	20.69	10.53	
milking vacuum	Mean (SD)	43.18 ±3.25	45.17 ±3.19	43.26 ±3.67	42.93 ±3.32	40.95 ±6.59	kPa
number of high temperature days	Mean (SD)	0.15 ±0.05	0.26 ±0.06	0.24 ±0.04	0.34 ±0.02	0.29 ±0.04	days
number of high wind days	Mean (SD)	0.07 ±0.08	0.03 ±0.06	0.11 ±0.14	0.18 ±0.12	0.09 ±0.1	days
number of low temperature days	Mean (SD)	0.3 ±0.07	0.21 ±0.06	0.16 ±0.05	0.13 ±0.02	0.14 ±0.04	days
number of milking places	Mean (SD)	3.91 ±2.21	3.35 ±1.85	6.95 ±7.44	6.93 ±6.38	5.64 ±2.62	n
pasture of dry cows	Percentage	75	47.83	48.89	6.9	36.84	
pasture of lactating cows	Percentage	58.33	39.13	53.33	3.45	38.6	
pasture of young stock	Percentage	91.67	95.65	75.56	37.93	64.91	
provision of concentrates: exact	Percentage	83.33	69.57	75.56	72.41	71.93	
provision of concentrates: manual	Percentage	16.67	30.43	15.56	13.79	21.05	
provision of concentrates: total mix ration	Percentage	0	0	4.44	13.79	7.02	
provision of supplementary concentrate: electronic feeder	Percentage	83.33	60.87	64.44	68.97	66.67	
provision of supplementary concentrate: exact	Percentage	0	8.7	11.11	3.45	5.26	
provision of supplementary concentrate: manual, two times a day	Percentage	0	21.74	11.11	6.9	17.54	
provision of supplementary concentrate: other	Percentage	16.67	8.7	13.33	20.69	10.53	
silo type: bunker silo	Percentage	33.33	39.13	60	86.21	70.18	
silo type: no silo	Percentage	8.33	4.35	20	0	8.77	
silo type: silage bales	Percentage	58.33	47.83	11.11	10.34	17.54	
standard deviation content of crude fibre for dry cows	Mean (SD)	30.35 ±17.16	25.86 ±16.39	23.14 ±9.02	27.93 ±13.43	26.98 ±12.95	
standard deviation content of crude fibre for lactating cows	Mean (SD)	12.59 ±3.57	14.94 ±5.81	15.45 ±6.36	15.79 ±6.43	15.7 ±5.31	
standard deviation for dietary proportion of concentrates for dry cows	Mean (SD)	0.08 ±0.03	0.07 ±0.05	0.06 ±0.04	0.07 ±0.03	0.08 ±0.04	
standard deviation for dietary proportion of concentrates for lactating cows	Mean (SD)	0.06 ±0.02	0.06 ±0.03	0.07 ±0.03	0.07 ±0.04	0.06 ±0.02	
standard deviation of yearly precipitation	Mean (SD)	6.31 ±1.03	5.69 ±0.87	6.56 ±1.65	5.77 ±0.82	6.37 ±1.12	mm

Table 6. Descriptive statistics for each cluster.*(continued)*

Variable	Statistic	Cluster 1	Cluster 2	Cluster 3	Cluster 4	Cluster 5	Unit
standard deviation of yearly relative humidity	Mean (SD)	11.54 ±2.06	11.41 ±1.27	11.69 ±1.56	10.85 ±1.05	11.62 ±1.14	%
standard deviation of yearly temperature	Mean (SD)	6.32 ±0.19	6.46 ±0.22	6.57 ±0.25	6.9 ±0.07	6.69 ±0.19	degree
type of milking stalls: herringbone parlour	Percentage	25	26.09	35.56	44.83	47.37	
type of milking stalls: milking robot	Percentage	25	8.7	4.44	10.34	7.02	
type of milking stalls: pipe milking	Percentage	16.67	26.09	24.44	0	3.51	
type of milking stalls: side-by-side	Percentage	25	4.35	6.67	13.79	8.77	
type of milking stalls: tandem type	Percentage	8.33	21.74	22.22	31.03	28.07	
used forage types in diet: corn silage, hay, pasture	Percentage	0	8.7	2.22	13.79	5.26	
used forage types in diet: field forage silage, grass silage, hay, corn silage	Percentage	8.33	4.35	11.11	31.03	14.04	
used forage types in diet: field forage silage, grass silage, hay, corn silage, pasture	Percentage	0	4.35	8.89	6.9	21.05	
used forage types in diet: grass silage, green forage, hay, pasture	Percentage	16.67	13.04	4.44	0	5.26	
used forage types in diet: grass silage, hay	Percentage	8.33	13.04	13.33	3.45	1.75	
used forage types in diet: grass silage, hay, pasture	Percentage	16.67	8.7	17.78	0	10.53	
used forage types in diet: other	Percentage	50	43.48	42.22	41.38	40.35	
walkway floor in free-stall for dry cows: concrete slits	Percentage	8.33	13.04	20	31.03	28.07	
walkway floor in free-stall for dry cows: other	Percentage	8.33	26.09	17.78	20.69	14.04	
walkway floor in free-stall for dry cows: rubber mats	Percentage	33.33	4.35	8.89	0	10.53	
walkway floor in free-stall for dry cows: solid concrete	Percentage	25	13.04	20	34.48	24.56	
walkway floor in free-stall for dry cows: solid concrete with slits	Percentage	8.33	17.39	11.11	3.45	12.28	
walkway floor in free-stall for lactating cows: concrete slits	Percentage	16.67	17.39	13.33	34.48	26.32	
walkway floor in free-stall for lactating cows: other	Percentage	16.67	26.09	20	17.24	15.79	
walkway floor in free-stall for lactating cows: rubber mats	Percentage	25	4.35	13.33	3.45	17.54	
walkway floor in free-stall for lactating cows: rubberised slits	Percentage	0	13.04	2.22	6.9	8.77	
walkway floor in free-stall for lactating cows: solid concrete	Percentage	16.67	8.7	22.22	31.03	17.54	
walkway floor in free-stall for lactating cows: solid concrete with slits	Percentage	8.33	4.35	6.67	6.9	12.28	
young stock are kept in a tie stall facility	Percentage	16.67	26.09	13.33	0	7.02	
young stock on alpine pasture	Percentage	83.33	91.3	42.22	3.45	24.56	

Table 7. Odds ratios for *anestrous*. ORs are obtained form the logistic regression model. Variables are listed in alphabetical order.

Variable	OR	CI.Low	CI.High	P-Value	R2	VIF
altitude	0.34	0.31	0.38	0.00	0.29	1.39
barn design: free-stall barn	0.65	0.55	0.78	0.00	0.22	1.05
barn design: outdoor climate house open front	3.20	2.68	3.82	0.00	0.24	1.22
beef index	0.82	0.76	0.89	0.00	0.19	1.03
body condition score	0.83	0.76	0.90	0.00	0.22	1.10
breeding value: milk yield	1.34	1.23	1.47	0.00	0.22	1.28
chest girth	1.17	1.06	1.28	0.00	0.22	1.32
claw trimming done by farmer	0.45	0.38	0.53	0.00	0.23	1.07
claw trimming frequency: once per year	1.29	1.02	1.63	0.03	0.20	1.24
claw trimming frequency: three times per year	1.88	1.51	2.36	0.00	0.20	1.38
claw trimming frequency: twice per year	0.57	0.48	0.68	0.00	0.20	1.10
concentrate dry matter intake	1.13	1.02	1.25	0.02	0.22	1.41
cubicle housing system: deep bed cubicles and slatted floors	0.77	0.63	0.94	0.01	0.22	1.15
diet: content of ash	0.65	0.58	0.72	0.00	0.23	1.07
diet: content of crude fat	1.14	1.05	1.23	0.00	0.22	1.10
diet: content of crude fibre	0.43	0.38	0.49	0.00	0.24	1.33
diet: content of degradable protein	0.84	0.76	0.92	0.00	0.22	1.06
diet: content of metabolisable energy	1.66	1.46	1.88	0.00	0.23	1.32
diet: content of nitrogen-free extracts	1.96	1.78	2.17	0.00	0.24	1.14
diet: content of organic matter	1.55	1.39	1.72	0.00	0.23	1.07
diet: content of undegraded protein	1.59	1.44	1.76	0.00	0.23	1.40
diet: content of utilizable protein	1.45	1.30	1.62	0.00	0.22	1.32
diet: content ruminal nitrogen balance	0.89	0.81	0.98	0.01	0.22	1.07
diet: total amount of crude fibre	0.85	0.77	0.93	0.00	0.22	1.21
diet: total amount of crude protein	1.45	1.31	1.60	0.00	0.22	1.49
diet: total amount of degradable protein	1.27	1.15	1.41	0.00	0.22	1.38
diet: total amount of ether extracts	1.44	1.32	1.57	0.00	0.23	1.38
diet: total amount of metabolisable energy	1.71	1.53	1.89	0.00	0.23	1.56
diet: total amount of net energy	1.75	1.57	1.94	0.00	0.23	1.58
diet: total amount of nitrogen-free extracts	1.95	1.77	2.16	0.00	0.24	1.51
diet: total amount of organic matter	1.68	1.51	1.86	0.00	0.23	1.53
diet: total amount of undegraded dietary protein	1.74	1.58	1.92	0.00	0.23	1.59
diet: total amount of utilizable protein	1.69	1.52	1.87	0.00	0.23	1.57
dietary proportion of concentrates	1.42	1.27	1.60	0.00	0.22	1.31
dietary proportion of concentrates for dry cows	0.88	0.81	0.95	0.00	0.22	1.06
dietary proportion of concentrates for lactating cows	1.59	1.42	1.78	0.00	0.23	1.43
dietary proportion of corn silage	2.05	1.88	2.23	0.00	0.26	1.18
dietary proportion of grass silage	0.61	0.56	0.67	0.00	0.23	1.16
dietary proportion of hay	0.85	0.74	0.98	0.03	0.22	1.08
dietary proportion of straw	2.24	1.88	2.66	0.00	0.23	1.15
dry cows group management: separate	0.83	0.70	0.99	0.03	0.22	1.13
dry cows group management: with lactating cows	1.36	1.15	1.62	0.00	0.22	1.09
energy balance	1.60	1.44	1.78	0.00	0.23	1.64
feed quality problematic	1.23	1.13	1.34	0.00	0.22	1.13
feeding group: lactating cows	1.35	1.08	1.68	0.01	0.22	1.16
fitness index	0.76	0.69	0.83	0.00	0.19	1.03
floor in open air area for young stock: no open-air areas	1.84	1.52	2.24	0.00	0.25	1.15
floor in open-air area for dry cows: no open-air areas	3.83	3.08	4.77	0.00	0.25	1.14
floor in open-air area for lactating cows: no open-air areas	2.95	2.48	3.52	0.00	0.25	1.08
floor in open-air area for young stock: solid concrete	0.72	0.59	0.87	0.00	0.24	1.08
floor in open-air areas for dry cows: solid concrete	0.22	0.17	0.28	0.00	0.25	1.13
floor in open-air areas for lactating cows: other	1.64	1.31	2.06	0.00	0.22	1.08
floor in open-air areas for lactating cows: solid concrete	0.22	0.18	0.27	0.00	0.26	1.02
floor in walkway of free-stall for young stock: concrete slits	0.77	0.63	0.93	0.01	0.22	1.23
floor in walkway of free-stall for young stock: solid concrete	0.47	0.39	0.58	0.00	0.23	1.23
forage dry matter intake	1.52	1.37	1.69	0.00	0.23	1.42
forage type: grass plus corn and grass products plus corn	2.39	1.85	3.07	0.00	0.22	1.07
forage type: mixed ration with concentrates	1.74	1.47	2.06	0.00	0.22	1.13
forage type: partial mixed ration	0.56	0.46	0.70	0.00	0.23	1.15

Table 7. Anestrous *(continued)*

Variable	OR	CI.Low	CI.High	P-Value	R2	VIF
forage type: sequentially fed forages	0.53	0.44	0.63	0.00	0.24	1.16
free-stall system for lactating cows: deep bed cubicle	0.71	0.57	0.89	0.00	0.22	1.08
free-stall system for young stock: other	0.67	0.54	0.84	0.00	0.22	1.06
herd size	1.37	1.26	1.48	0.00	0.22	1.30
litter in free-stall for lactating cows: chopped straw	1.51	1.25	1.83	0.00	0.21	1.14
litter in free-stall for lactating cows: long straw	0.61	0.46	0.80	0.00	0.21	1.05
main breed: Fleckvieh	3.01	2.01	4.51	0.00	0.22	5.99
manure removal in free-stall for young stock: scraper	0.53	0.41	0.67	0.00	0.23	1.12
manure removal in free-stall for dry cows: other	1.39	1.17	1.65	0.00	0.22	1.14
manure removal in free-stall for dry cows: scraper	0.68	0.57	0.81	0.00	0.22	1.05
manure removal in free-stall for lactating cows: scraper	1.39	1.17	1.65	0.00	0.22	1.11
manure removal in free-stall for lactating cows: slits	0.64	0.53	0.77	0.00	0.22	1.03
manure removal in free-stall for young stock: other	1.37	1.15	1.63	0.00	0.22	1.08
manure removal: slurry with perforated flooring	0.59	0.49	0.72	0.00	0.22	1.04
manure removal: slurry with solid flooring	1.35	1.15	1.58	0.00	0.22	1.02
mean content of crude fibre for lactating cows	0.32	0.28	0.36	0.00	0.27	1.47
mean yearly precipitation	0.50	0.44	0.57	0.00	0.23	1.05
mean yearly temperature	2.99	2.60	3.43	0.00	0.27	1.35
milk index	1.20	1.10	1.32	0.00	0.22	1.34
milking vacuum	0.74	0.69	0.79	0.00	0.18	1.01
muscularity score	0.61	0.56	0.68	0.00	0.23	1.37
number of high temperature days	2.55	2.30	2.84	0.00	0.27	1.12
number of high wind days	1.29	1.19	1.40	0.00	0.22	1.30
number of low temperature days	0.61	0.54	0.68	0.00	0.23	1.31
number of milking places	1.45	1.37	1.53	0.00	0.21	1.14
pasture of dry cows	0.59	0.48	0.72	0.00	0.22	1.07
pasture of lactating cows	0.55	0.44	0.68	0.00	0.22	1.13
pasture of young stock	0.56	0.45	0.69	0.00	0.22	1.60
problematic feed quality	1.52	1.19	1.93	0.00	0.19	1.05
provision of concentrates: exact	0.31	0.26	0.37	0.00	0.24	1.05
provision of supplementary concentrate: electronic feeder	0.39	0.33	0.47	0.00	0.23	1.06
provision of supplementary concentrate: other	3.65	3.05	4.36	0.00	0.24	1.10
ration type: feedstuffs sequentially fed	0.33	0.27	0.40	0.00	0.24	1.08
ration type: partial mixed ration	1.34	1.13	1.59	0.00	0.22	1.12
silo type: bunker silo	2.23	1.82	2.73	0.00	0.23	1.12
silo type: silage bales	0.48	0.39	0.60	0.00	0.23	1.19
standard deviation content of crude fibre for lactating cows	0.89	0.82	0.97	0.01	0.22	1.11
standard deviation for dietary proportion of concentrates for dry cows	1.67	1.53	1.81	0.00	0.24	1.12
standard deviation for dietary proportion of concentrates for lactating cows	0.91	0.84	0.99	0.03	0.22	1.08
standard deviation of yearly relative humidity	1.24	1.14	1.35	0.00	0.22	1.15
standard deviation of yearly temperature	3.55	3.10	4.06	0.00	0.29	1.06
test-day fat yield percentage	0.83	0.76	0.90	0.00	0.22	1.07
test-day lactose content	1.39	1.24	1.57	0.00	0.22	1.27
test-day milk yield	1.52	1.37	1.70	0.00	0.23	2.07
test-day protein yield percentage	0.71	0.64	0.80	0.00	0.22	1.53
total dry matter intake	1.63	1.47	1.80	0.00	0.23	1.52
type of milking stalls: herringbone parlour	1.58	1.33	1.86	0.00	0.22	1.11
type of milking stalls: tandem type	0.71	0.56	0.89	0.00	0.22	1.07
used forage types in diet: field forage silage, grass silage, hay, corn silage, pasture	1.72	1.40	2.13	0.00	0.22	1.02
used forage types in diet: other	1.69	1.43	1.99	0.00	0.23	1.06
walkway floor in free-stall for dry cows: solid concrete	2.08	1.76	2.45	0.00	0.22	1.03
walkway floor in free-stall for lactating cows: solid concrete	1.76	1.48	2.11	0.00	0.22	1.07
young stock on alpine pasture	0.21	0.17	0.26	0.00	0.26	1.29

Table 8. Odds ratios for *acute mastitis*. ORs are obtained form the logistic regression model. Variables are listed in alphabetical order.

Variable	OR	CI.Low	CI.High	P-Value	R2	VIF
altitude	0.57	0.52	0.63	0.00	0.11	1.29
automated milking switch-off	0.72	0.60	0.87	0.00	0.09	1.08
barn design: free-stall barn	0.80	0.69	0.94	0.01	0.09	1.03
barn design: outdoor climate house closed	1.22	1.03	1.46	0.02	0.09	1.07
barn design: outdoor climate house open front	1.73	1.45	2.06	0.00	0.09	1.14
beef index	0.90	0.84	0.97	0.01	0.09	1.01
body condition score	0.89	0.82	0.97	0.01	0.09	1.08
breeding value: milk yield	1.42	1.30	1.54	0.00	0.10	1.34
chest girth	1.12	1.02	1.22	0.02	0.09	1.36
claw trimming done by farmer	0.54	0.47	0.64	0.00	0.10	1.02
claw trimming frequency: three times per year	1.37	1.12	1.67	0.00	0.09	1.16
claw trimming frequency: twice per year	0.79	0.67	0.92	0.00	0.09	1.03
cubicle housing system: deep bed cubicles and solid floors	0.74	0.64	0.87	0.00	0.09	1.17
cubicle housing system: other	1.87	1.43	2.45	0.00	0.09	1.09
diet: content of ash	0.85	0.78	0.92	0.00	0.09	1.07
diet: content of crude fibre	0.85	0.77	0.94	0.00	0.09	1.34
diet: content of crude protein	0.89	0.82	0.97	0.01	0.09	1.19
diet: content of degradable protein	0.80	0.73	0.87	0.00	0.09	1.09
diet: content of nitrogen-free extracts	1.26	1.16	1.37	0.00	0.09	1.12
diet: content of organic matter	1.18	1.08	1.28	0.00	0.09	1.07
diet: content of undegraded protein	1.24	1.13	1.35	0.00	0.09	1.41
diet: content ruminal nitrogen balance	0.84	0.77	0.91	0.00	0.09	1.07
diet: total amount of ether extracts	1.13	1.03	1.23	0.01	0.09	1.37
diet: total amount of metabolisable energy	1.19	1.08	1.31	0.00	0.09	1.49
diet: total amount of net energy	1.20	1.09	1.32	0.00	0.09	1.51
diet: total amount of nitrogen-free extracts	1.29	1.18	1.41	0.00	0.09	1.40
diet: total amount of organic matter	1.22	1.11	1.34	0.00	0.09	1.44
diet: total amount of undegraded dietary protein	1.29	1.18	1.41	0.00	0.09	1.54
diet: total amount of utilizable protein	1.21	1.10	1.33	0.00	0.09	1.52
diet: total ruminal nitrogen balance	0.86	0.80	0.93	0.00	0.09	1.09
dietary proportion of clover	0.66	0.49	0.87	0.00	0.09	1.05
dietary proportion of concentrates	1.13	1.03	1.25	0.01	0.09	1.46
dietary proportion of concentrates for lactating cows	1.30	1.18	1.43	0.00	0.09	1.47
dietary proportion of corn silage	1.45	1.35	1.56	0.00	0.10	1.11
dietary proportion of grass silage	0.90	0.83	0.97	0.01	0.09	1.08
dietary proportion of straw	1.21	1.04	1.42	0.02	0.09	1.11
dry cows group management: separate	0.76	0.65	0.88	0.00	0.09	1.10
dry cows group management: with lactating cows	1.22	1.05	1.43	0.01	0.09	1.05
energy balance	1.18	1.07	1.30	0.00	0.09	1.51
farm organically managed	0.71	0.56	0.90	0.01	0.09	1.19
feed quality problematic	1.27	1.18	1.36	0.00	0.09	1.08
feeding group: lactating cows	1.43	1.19	1.71	0.00	0.09	1.10
fitness index	0.72	0.66	0.78	0.00	0.10	1.02
floor in open air area for young stock: no open-air areas	1.25	1.05	1.49	0.01	0.09	1.12
floor in open-air area for dry cows: no open-air areas	1.28	1.09	1.50	0.00	0.09	1.08
floor in open-air area for lactating cows: no open-air areas	1.34	1.15	1.56	0.00	0.09	1.10
floor in open-air areas for dry cows: other	1.56	1.20	2.02	0.00	0.09	1.03
floor in open-air areas for dry cows: solid concrete	0.65	0.55	0.78	0.00	0.09	1.07
floor in open-air areas for lactating cows: other	1.58	1.26	1.97	0.00	0.09	1.05
floor in open-air areas for lactating cows: solid concrete	0.61	0.52	0.72	0.00	0.10	1.06
floor in walkway of free-stall for young stock: concrete slits	1.39	1.17	1.64	0.00	0.10	1.13
floor in walkway of free-stall for young stock: solid concrete	0.67	0.56	0.81	0.00	0.10	1.14
forage dry matter intake	1.17	1.07	1.29	0.00	0.09	1.50
forage type: grass and grass products only	0.49	0.38	0.64	0.00	0.09	1.06
forage type: grass plus corn and grass products plus corn	1.69	1.33	2.15	0.00	0.09	1.04
forage type: mixed ration with concentrates	1.28	1.09	1.50	0.00	0.09	1.15
forage type: other	0.69	0.53	0.89	0.00	0.09	1.01
forage type: partial mixed ration	0.82	0.69	0.97	0.02	0.09	1.09
forage type: sequentially fed forages	0.73	0.62	0.86	0.00	0.10	1.19

Table 8. Acute mastitis *(continued)*

Variable	OR	CI.Low	CI.High	P-Value	R2	VIF
forage type: total mixed ration	3.22	2.58	4.03	0.00	0.11	1.22
free-stall system for dry cows: deep bed cubicle	0.72	0.62	0.84	0.00	0.09	1.02
free-stall system for dry cows: high bed cubicle	1.57	1.24	1.98	0.00	0.09	1.11
free-stall system for lactating cows: deep bed cubicle	0.62	0.51	0.75	0.00	0.09	1.06
free-stall system for lactating cows: high bed cubicle	1.52	1.16	1.98	0.00	0.09	1.12
free-stall system for lactating cows: other	1.56	1.23	1.97	0.00	0.09	1.03
free-stall system for young stock: deep bed cubicle	0.47	0.36	0.61	0.00	0.11	1.03
free-stall system for young stock: deep litter	0.78	0.62	0.98	0.03	0.10	1.02
free-stall system for young stock: high bed cubicle	1.88	1.60	2.21	0.00	0.11	1.03
herd size	1.21	1.12	1.30	0.00	0.09	1.19
litter in free-stall for lactating cows: long straw	0.79	0.64	0.98	0.04	0.09	1.07
main breed: Fleckvieh	2.81	1.94	4.08	0.00	0.09	5.56
main breed: Holstein	0.32	0.21	0.47	0.00	0.09	3.42
manure removal in free-stall for young stock: scraper	0.74	0.60	0.91	0.00	0.09	1.03
manure removal in free-stall for dry cows: scraper	0.83	0.71	0.97	0.02	0.09	1.04
manure removal in free-stall for dry cows: slits	1.45	1.22	1.73	0.00	0.09	1.06
manure removal in free-stall for young stock: slits	1.34	1.13	1.57	0.00	0.10	1.08
manure removal: solid manure	1.37	1.06	1.77	0.02	0.09	1.05
mean content of crude fibre for dry cows	0.92	0.85	0.99	0.03	0.09	1.08
mean content of crude fibre for lactating cows	0.73	0.67	0.81	0.00	0.09	1.36
mean yearly precipitation	0.57	0.51	0.63	0.00	0.11	1.05
mean yearly relative humidity	1.36	1.23	1.49	0.00	0.09	1.14
mean yearly temperature	1.57	1.41	1.74	0.00	0.10	1.18
milk index	1.27	1.17	1.39	0.00	0.09	1.38
milking take off: present	0.83	0.71	0.97	0.02	0.09	1.04
milking unit removal: present, including post-milking technology	1.82	1.48	2.23	0.00	0.09	1.06
milking vacuum	1.32	1.18	1.47	0.00	0.09	1.14
muscularity score	0.71	0.65	0.77	0.00	0.10	1.24
number of high temperature days	1.55	1.42	1.70	0.00	0.10	1.08
number of high wind days	1.19	1.11	1.27	0.00	0.09	1.15
number of low temperature days	0.79	0.72	0.87	0.00	0.09	1.15
number of milking places	1.17	1.09	1.25	0.00	0.09	1.08
pasture of dry cows	0.74	0.63	0.88	0.00	0.09	1.05
pasture of lactating cows	0.72	0.60	0.86	0.00	0.09	1.13
pasture of young stock	0.70	0.59	0.83	0.00	0.09	1.23
problematic feed quality	1.34	1.09	1.66	0.01	0.08	1.02
provision of concentrates: exact	0.46	0.39	0.55	0.00	0.10	1.04
provision of concentrates: manual	1.31	1.02	1.69	0.04	0.09	1.14
provision of concentrates: total mix ration	3.37	2.70	4.20	0.00	0.11	1.19
provision of supplementary concentrate: electronic feeder	0.52	0.45	0.61	0.00	0.10	1.02
provision of supplementary concentrate: other	2.89	2.41	3.47	0.00	0.11	1.10
ration type: feedstuffs sequentially fed	0.66	0.56	0.77	0.00	0.09	1.10
ration type: partial mixed ration	1.19	1.02	1.39	0.03	0.09	1.12
silo type: bunker silo	2.14	1.75	2.62	0.00	0.10	1.06
silo type: silage bales	0.57	0.46	0.71	0.00	0.10	1.13
standard deviation for dietary proportion of concentrates for dry cows	1.34	1.24	1.44	0.00	0.10	1.10
standard deviation for dietary proportion of concentrates for lactating cows	0.89	0.82	0.96	0.00	0.09	1.07
standard deviation of yearly precipitation	0.91	0.84	0.98	0.02	0.09	1.05
standard deviation of yearly relative humidity	0.83	0.76	0.90	0.00	0.09	1.05
standard deviation of yearly temperature	2.03	1.84	2.25	0.00	0.12	1.04
test-day energy corrected milk daily yield	0.88	0.81	0.95	0.00	0.09	1.13
test-day lactose content	0.88	0.81	0.95	0.00	0.09	1.33
test-day milk yield	1.27	1.14	1.40	0.00	0.09	2.11
test-day somatic cell count	1.73	1.61	1.85	0.00	0.12	1.12
total dry matter intake	1.25	1.14	1.37	0.00	0.09	1.44
type of milking stalls: herringbone parlour	1.20	1.03	1.41	0.02	0.09	1.10
type of milking stalls: milking robot	0.52	0.40	0.67	0.00	0.09	1.04
type of milking stalls: side-by-side	1.28	1.03	1.59	0.03	0.09	1.05
used forage types in diet: field forage silage, grass silage, hay, corn silage	1.41	1.17	1.70	0.00	0.09	1.03
walkway floor in free-stall for dry cows: concrete slits	1.65	1.37	1.97	0.00	0.09	1.09

Table 8. Acute mastitis *(continued)*

Variable	OR	CI.Low	CI.High	P-Value	R2	VIF
walkway floor in free-stall for dry cows: other	0.48	0.38	0.60	0.00	0.10	1.01
walkway floor in free-stall for dry cows: solid concrete	1.47	1.26	1.73	0.00	0.09	1.02
walkway floor in free-stall for dry cows: solid concrete with slits	0.49	0.38	0.65	0.00	0.09	1.04
walkway floor in free-stall for lactating cows: concrete slits	1.39	1.15	1.67	0.00	0.09	1.02
walkway floor in free-stall for lactating cows: other	0.62	0.50	0.76	0.00	0.10	1.08
walkway floor in free-stall for lactating cows: rubber mats	1.53	1.26	1.85	0.00	0.10	1.04
walkway floor in free-stall for lactating cows: solid concrete	1.23	1.04	1.46	0.02	0.09	1.03
young stock on alpine pasture	0.33	0.27	0.41	0.00	0.11	1.23

Table 9. Odds ratios for *chronic mastitis*. ORs are obtained form the logistic regression model. Variables are listed in alphabetical order.

Variable	OR	CI.Low	CI.High	P-Value	R2	VIF
altitude	0.63	0.54	0.72	0.00	0.08	1.12
barn design: outdoor climate house closed	2.92	2.29	3.73	0.00	0.09	1.07
body condition score	0.84	0.74	0.96	0.01	0.07	1.07
body weight	1.33	1.15	1.54	0.00	0.07	1.60
breeding value: milk yield	1.47	1.28	1.68	0.00	0.08	1.41
chest girth	1.53	1.35	1.74	0.00	0.08	1.36
claw trimming done by farmer	0.51	0.40	0.65	0.00	0.08	1.02
concentrate dry matter intake	1.29	1.13	1.47	0.00	0.07	1.44
cubicle housing system: deep bed cubicles and slatted floors	2.07	1.59	2.69	0.00	0.08	1.19
cubicle housing system: deep bed cubicles and solid floors	0.64	0.50	0.83	0.00	0.07	1.09
diet: content of ash	0.84	0.73	0.95	0.01	0.07	1.10
diet: content of crude fat	1.33	1.19	1.49	0.00	0.08	1.12
diet: content of organic matter	1.20	1.05	1.36	0.01	0.07	1.10
diet: content of undegraded protein	1.46	1.27	1.69	0.00	0.08	1.45
diet: total amount of crude fibre	1.35	1.19	1.52	0.00	0.08	1.19
diet: total amount of crude protein	1.20	1.03	1.40	0.02	0.07	1.48
diet: total amount of ether extracts	1.42	1.25	1.62	0.00	0.08	1.34
diet: total amount of metabolisable energy	1.22	1.04	1.42	0.01	0.07	1.48
diet: total amount of net energy	1.20	1.03	1.40	0.02	0.07	1.50
diet: total amount of nitrogen-free extracts	1.20	1.03	1.38	0.02	0.07	1.40
diet: total amount of organic matter	1.30	1.12	1.50	0.00	0.07	1.41
diet: total amount of undegraded dietary protein	1.45	1.26	1.68	0.00	0.08	1.56
diet: total amount of utilizable protein	1.28	1.10	1.49	0.00	0.07	1.51
dietary proportion of corn silage	1.40	1.24	1.58	0.00	0.08	1.08
dietary proportion of hay	0.62	0.50	0.76	0.00	0.08	1.06
dietary proportion of straw	1.74	1.36	2.24	0.00	0.07	1.09
dry cows group management: separate	0.70	0.55	0.90	0.00	0.07	1.05
dry cows group management: with lactating cows	1.48	1.16	1.88	0.00	0.07	1.04
energy balance	1.33	1.13	1.57	0.00	0.07	1.63
feed quality problematic	1.55	1.41	1.71	0.00	0.09	1.03
feeding group: lactating cows	2.47	1.82	3.34	0.00	0.08	1.05
fitness index	0.67	0.59	0.76	0.00	0.08	1.04
floor in open-air area for dry cows: no open-air areas	0.72	0.56	0.92	0.01	0.07	1.09
floor in open-air areas for dry cows: solid concrete	0.56	0.42	0.74	0.00	0.07	1.09
floor in open-air areas for lactating cows: solid concrete	0.61	0.47	0.78	0.00	0.07	1.10
floor in walkway of free-stall for young stock: concrete slits	2.30	1.74	3.05	0.00	0.09	1.09
forage type: sequentially fed forages	0.65	0.50	0.84	0.00	0.07	1.19
forage type: year-round silage (with corn silage)	2.28	1.73	3.01	0.00	0.08	1.09
free-stall system for dry cows: deep bed cubicle	0.58	0.45	0.75	0.00	0.08	1.06
free-stall system for dry cows: other	2.25	1.72	2.94	0.00	0.09	1.09
free-stall system for lactating cows: deep bed cubicle	0.29	0.23	0.38	0.00	0.10	1.08
herd size	1.59	1.42	1.78	0.00	0.09	1.19
main breed: Brown Swiss	3.22	1.70	6.08	0.00	0.07	6.29
main breed: Fleckvieh	2.42	1.41	4.15	0.00	0.07	5.10
manure removal in free-stall for dry cows: other	0.60	0.45	0.80	0.00	0.08	1.05
manure removal in free-stall for dry cows: scraper	1.74	1.36	2.24	0.00	0.08	1.08
manure removal in free-stall for lactating cows: scraper	1.49	1.17	1.89	0.00	0.07	1.05
manure removal in free-stall for young stock: slits	1.91	1.45	2.50	0.00	0.08	1.07
mean yearly precipitation	0.43	0.36	0.51	0.00	0.11	1.06
mean yearly relative humidity	1.65	1.41	1.92	0.00	0.08	1.12
mean yearly temperature	1.60	1.35	1.89	0.00	0.08	1.13
milk index	1.45	1.26	1.66	0.00	0.08	1.44
milking unit removal: none	0.53	0.42	0.68	0.00	0.08	1.02
milking unit removal: present, including post-milking technology	3.30	2.52	4.32	0.00	0.09	1.05
milking vacuum	2.96	2.48	3.53	0.00	0.12	1.18
muscularity score	0.75	0.66	0.85	0.00	0.07	1.20
number of high temperature days	1.89	1.62	2.20	0.00	0.09	1.06
number of milking places	1.14	1.04	1.25	0.01	0.07	1.06
pasture of lactating cows	0.38	0.28	0.52	0.00	0.08	1.13

Table 9. Chronic mastitis *(continued)*

Variable	OR	CI.Low	CI.High	P-Value	R2	VIF
pasture of young stock	0.46	0.36	0.60	0.00	0.08	1.17
provision of concentrates: exact	0.36	0.28	0.46	0.00	0.09	1.06
provision of supplementary concentrate: electronic feeder	0.39	0.31	0.50	0.00	0.09	1.03
provision of supplementary concentrate: other	5.22	4.01	6.78	0.00	0.11	1.07
ration type: feedstuffs sequentially fed	0.52	0.40	0.67	0.00	0.08	1.09
silo type: bunker silo	5.82	3.59	9.43	0.00	0.09	1.01
standard deviation content of crude fibre for dry cows	1.15	1.04	1.29	0.01	0.07	1.03
standard deviation for dietary proportion of concentrates for dry cows	1.72	1.54	1.93	0.00	0.10	1.12
standard deviation of yearly precipitation	0.67	0.59	0.76	0.00	0.08	1.07
standard deviation of yearly relative humidity	0.75	0.66	0.86	0.00	0.07	1.05
standard deviation of yearly temperature	2.65	2.25	3.12	0.00	0.12	1.04
test-day fat yield percentage	1.15	1.02	1.29	0.02	0.07	1.07
test-day milk yield	1.38	1.16	1.63	0.00	0.07	2.24
test-day somatic cell count	2.13	1.91	2.37	0.00	0.13	1.11
total dry matter intake	1.28	1.11	1.48	0.00	0.07	1.41
type of milking stalls: herringbone parlour	2.69	2.09	3.45	0.00	0.09	1.06
used forage types in diet: field forage silage, grass silage, hay, corn silage	2.03	1.56	2.66	0.00	0.08	1.05
used forage types in diet: other	1.46	1.15	1.86	0.00	0.07	1.04
waist circumference	1.35	1.17	1.55	0.00	0.07	1.33
walkway floor in free-stall for lactating cows: concrete slits	1.46	1.10	1.93	0.01	0.07	1.01
walkway floor in free-stall for lactating cows: rubber mats	1.96	1.49	2.57	0.00	0.08	1.03

Table 10. Odds ratios for *ketosis*. ORs are obtained form the logistic regression model. Variables are listed in alphabetical order.

Variable	OR	CI.Low	CI.High	P-Value	R2	VIF
altitude	0.68	0.60	0.78	0.00	0.09	1.16
automated milking switch-off	0.58	0.44	0.76	0.00	0.09	1.08
beef index	0.86	0.76	0.98	0.02	0.14	1.02
body condition score	1.73	1.52	1.97	0.00	0.11	1.08
body weight	1.87	1.62	2.16	0.00	0.11	1.62
breeding value: milk yield	1.25	1.10	1.43	0.00	0.08	1.32
chest girth	1.87	1.66	2.11	0.00	0.12	1.29
claw trimming frequency: once per year	1.64	1.21	2.23	0.00	0.08	1.20
concentrate dry matter intake	0.74	0.64	0.86	0.00	0.09	1.34
diet: content of ash	0.85	0.74	0.97	0.02	0.08	1.06
diet: content of crude fibre	1.33	1.15	1.54	0.00	0.09	1.26
diet: content of crude protein	0.76	0.67	0.87	0.00	0.09	1.20
diet: content of degradable protein	0.76	0.66	0.86	0.00	0.09	1.09
diet: content of metabolisable energy	0.68	0.59	0.79	0.00	0.09	1.32
diet: content of organic matter	1.18	1.03	1.35	0.02	0.08	1.06
diet: content of utilizable protein	0.71	0.61	0.83	0.00	0.09	1.38
diet: content ruminal nitrogen balance	0.84	0.74	0.96	0.01	0.08	1.07
diet: total amount of ash	0.56	0.49	0.65	0.00	0.10	1.15
diet: total amount of crude fibre	0.66	0.58	0.76	0.00	0.09	1.24
diet: total amount of crude protein	0.53	0.46	0.62	0.00	0.10	1.37
diet: total amount of degradable protein	0.52	0.45	0.60	0.00	0.11	1.33
diet: total amount of ether extracts	0.61	0.52	0.71	0.00	0.10	1.32
diet: total amount of metabolisable energy	0.51	0.44	0.60	0.00	0.11	1.38
diet: total amount of net energy	0.51	0.44	0.60	0.00	0.11	1.38
diet: total amount of nitrogen-free extracts	0.58	0.50	0.68	0.00	0.10	1.34
diet: total amount of organic matter	0.54	0.47	0.63	0.00	0.10	1.37
diet: total amount of undegraded dietary protein	0.67	0.57	0.78	0.00	0.09	1.44
diet: total amount of utilizable protein	0.52	0.45	0.60	0.00	0.11	1.40
diet: total ruminal nitrogen balance	0.81	0.71	0.91	0.00	0.09	1.09
dietary proportion of concentrates	0.70	0.61	0.81	0.00	0.09	1.32
dietary proportion of corn silage	1.25	1.11	1.40	0.00	0.09	1.11
energy balance	0.53	0.47	0.59	0.00	0.12	1.16
feed quality problematic	1.29	1.16	1.44	0.00	0.09	1.04
feeding group: lactating cows	1.36	1.04	1.78	0.03	0.08	1.09
fitness index	0.77	0.67	0.88	0.00	0.14	1.02
floor in open-air areas for lactating cows: other	1.92	1.39	2.63	0.00	0.09	1.05
floor in open-air areas for lactating cows: solid concrete	0.62	0.48	0.79	0.00	0.09	1.06
floor in walkway of free-stall for young stock: concrete slits	1.77	1.34	2.33	0.00	0.09	1.14
floor in walkway of free-stall for young stock: solid concrete	0.54	0.39	0.74	0.00	0.09	1.12
forage dry matter intake	0.62	0.54	0.72	0.00	0.09	1.33
forage type: mixed ration with concentrates	1.33	1.03	1.70	0.03	0.08	1.14
forage type: partial mixed ration	0.56	0.42	0.74	0.00	0.09	1.09
forage type: total mixed ration	4.83	3.56	6.55	0.00	0.11	1.16
forage type: year-round silage (with corn silage)	1.47	1.08	2.00	0.02	0.08	1.06
free-stall system for young stock: other	2.25	1.69	3.01	0.00	0.08	1.10
herd size	1.46	1.31	1.63	0.00	0.10	1.18
litter in free-stall for lactating cows: chopped straw	1.94	1.48	2.54	0.00	0.09	1.10
litter in free-stall for lactating cows: other	0.66	0.48	0.90	0.01	0.08	1.11
manure removal in free-stall for dry cows: other	0.64	0.49	0.84	0.00	0.09	1.05
manure removal in free-stall for dry cows: slits	1.39	1.05	1.83	0.02	0.08	1.06
manure removal in free-stall for lactating cows: scraper	1.56	1.22	2.00	0.00	0.09	1.07
manure removal in free-stall for young stock: slits	1.83	1.40	2.39	0.00	0.08	1.08
mean content of crude fibre for lactating cows	0.84	0.72	0.97	0.02	0.08	1.37
mean yearly precipitation	0.50	0.42	0.59	0.00	0.11	1.07
mean yearly relative humidity	1.40	1.21	1.62	0.00	0.09	1.12
mean yearly temperature	1.33	1.14	1.53	0.00	0.09	1.11
milking unit removal: none	0.73	0.58	0.93	0.01	0.08	1.02
milking unit removal: present, including post-milking technology	2.12	1.56	2.88	0.00	0.09	1.06
number of high temperature days	1.43	1.25	1.64	0.00	0.09	1.06

Table 10. Ketosis *(continued)*

Variable	OR	CI.Low	CI.High	P-Value	R2	VIF
number of high wind days	1.48	1.33	1.64	0.00	0.10	1.18
pasture of dry cows	0.56	0.43	0.75	0.00	0.09	1.06
pasture of lactating cows	0.46	0.34	0.62	0.00	0.09	1.11
pasture of young stock	0.68	0.53	0.88	0.00	0.08	1.18
problematic feed quality	1.49	1.06	2.11	0.02	0.14	1.02
provision of concentrates: exact	0.38	0.30	0.49	0.00	0.10	1.03
provision of concentrates: total mix ration	5.04	3.72	6.81	0.00	0.11	1.14
provision of supplementary concentrate: electronic feeder	0.52	0.41	0.66	0.00	0.09	1.02
provision of supplementary concentrate: other	3.37	2.57	4.41	0.00	0.10	1.08
ration type: feedstuffs sequentially fed	0.68	0.53	0.87	0.00	0.08	1.10
ration type: partial mixed ration	0.62	0.48	0.80	0.00	0.09	1.10
silo type: bunker silo	1.86	1.38	2.52	0.00	0.09	1.03
standard deviation for dietary proportion of concentrates for dry cows	1.47	1.31	1.65	0.00	0.09	1.10
standard deviation of yearly precipitation	0.69	0.61	0.79	0.00	0.09	1.06
standard deviation of yearly relative humidity	0.87	0.77	0.98	0.03	0.08	1.06
standard deviation of yearly temperature	1.88	1.63	2.17	0.00	0.11	1.06
test-day fat yield percentage	1.54	1.39	1.70	0.00	0.10	1.01
test-day fat-protein ratio	1.43	1.24	1.65	0.00	0.11	1.03
test-day lactose content	0.62	0.56	0.69	0.00	0.09	1.30
test-day protein yield percentage	1.43	1.25	1.65	0.00	0.08	1.24
test-day somatic cell count	1.63	1.46	1.82	0.00	0.10	1.11
TMI	0.86	0.75	0.98	0.03	0.08	1.44
total dry matter intake	0.53	0.46	0.61	0.00	0.11	1.38
type of milking stalls: herringbone parlour	1.56	1.22	1.98	0.00	0.09	1.07
used forage types in diet: field forage silage, grass silage, hay, corn silage	2.33	1.77	3.07	0.00	0.13	1.05
walkway floor in free-stall for dry cows: concrete slits	1.55	1.16	2.06	0.00	0.09	1.08
walkway floor in free-stall for lactating cows: solid concrete	1.53	1.18	2.00	0.00	0.09	1.05
young stock on alpine pasture	0.25	0.18	0.34	0.00	0.11	1.16

Table 11. Odds ratios for *lameness*. ORs are obtained form the logistic regression model. Variables are listed in alphabetical order.

Variable	OR	CI.Low	CI.High	P-Value	R2	VIF
age at first calving	1.11	1.06	1.15	0.00	0.14	1.08
altitude	0.70	0.67	0.73	0.00	0.16	1.18
automated milking switch-off	0.69	0.63	0.77	0.00	0.14	1.06
barn design: free-stall barn	1.24	1.15	1.35	0.00	0.14	1.03
barn design: other	0.32	0.27	0.38	0.00	0.15	1.05
barn design: outdoor climate house open front	1.32	1.19	1.47	0.00	0.14	1.13
body condition score	0.69	0.66	0.72	0.00	0.16	1.12
body weight	0.84	0.80	0.89	0.00	0.15	1.74
chest girth	1.27	1.21	1.33	0.00	0.15	1.38
claw trimming done by farmer	0.79	0.72	0.86	0.00	0.14	1.02
claw trimming frequency: once per year	0.80	0.71	0.89	0.00	0.14	1.13
claw trimming frequency: only for lame animals	0.82	0.68	0.99	0.04	0.14	1.04
claw trimming frequency: three times per year	1.20	1.07	1.35	0.00	0.14	1.10
concentrate dry matter intake	0.82	0.78	0.86	0.00	0.15	1.34
cubicle housing system: deep bed cubicles and slatted floors	0.89	0.81	0.99	0.03	0.14	1.14
cubicle housing system: deep bed cubicles and solid floors	0.79	0.73	0.86	0.00	0.14	1.08
cubicle housing system: high bed cubicles and slatted floors	1.97	1.73	2.25	0.00	0.15	1.08
cubicle housing system: high bed cubicles and solid floors	1.43	1.25	1.63	0.00	0.14	1.10
cubicle housing system: other	0.74	0.62	0.89	0.00	0.14	1.03
diet: content of ash	1.09	1.05	1.14	0.00	0.14	1.09
diet: content of crude fat	1.11	1.06	1.16	0.00	0.14	1.12
diet: content of crude fibre	0.74	0.70	0.78	0.00	0.15	1.39
diet: content of crude protein	1.17	1.12	1.23	0.00	0.15	1.15
diet: content of nitrogen-free extracts	1.05	1.00	1.10	0.03	0.14	1.15
diet: content of organic matter	0.92	0.88	0.96	0.00	0.14	1.09
diet: content of undegraded protein	1.49	1.41	1.56	0.00	0.16	1.52
diet: content of utilizable protein	1.27	1.21	1.34	0.00	0.15	1.48
diet: content ruminal nitrogen balance	1.09	1.05	1.14	0.00	0.14	1.05
diet: total amount of ash	1.13	1.09	1.18	0.00	0.14	1.09
diet: total amount of crude fibre	0.82	0.78	0.86	0.00	0.15	1.20
diet: total amount of crude protein	1.17	1.11	1.23	0.00	0.14	1.48
diet: total amount of degradable protein	1.08	1.03	1.13	0.00	0.14	1.36
diet: total amount of ether extracts	1.13	1.08	1.19	0.00	0.14	1.43
diet: total amount of metabolisable energy	1.06	1.01	1.12	0.03	0.14	1.56
diet: total amount of net energy	1.07	1.02	1.13	0.01	0.14	1.57
diet: total amount of nitrogen-free extracts	1.08	1.02	1.14	0.01	0.14	1.49
diet: total amount of undegraded dietary protein	1.37	1.30	1.44	0.00	0.15	1.65
diet: total amount of utilizable protein	1.14	1.08	1.21	0.00	0.14	1.60
diet: total ruminal nitrogen balance	1.10	1.06	1.15	0.00	0.14	1.07
dietary proportion of clover	0.60	0.52	0.70	0.00	0.15	1.04
dietary proportion of concentrates	1.32	1.25	1.39	0.00	0.15	1.49
dietary proportion of concentrates for lactating cows	1.25	1.19	1.32	0.00	0.15	1.44
dietary proportion of corn silage	1.34	1.28	1.39	0.00	0.15	1.18
dietary proportion of grass silage	0.93	0.89	0.97	0.00	0.14	1.10
dietary proportion of green forage	0.87	0.76	0.99	0.03	0.14	1.21
dietary proportion of straw	1.13	1.03	1.23	0.01	0.14	1.12
dry cows group management: with lactating cows	1.22	1.12	1.32	0.00	0.14	1.03
dry cows group management: with young stock	0.42	0.34	0.52	0.00	0.15	1.03
dry cows on alpine pasture	0.53	0.45	0.63	0.00	0.15	1.18
dry matter	0.94	0.88	0.99	0.03	0.18	1.31
farm organically managed	0.63	0.56	0.71	0.00	0.15	1.25
feed quality problematic	1.45	1.39	1.50	0.00	0.16	1.04
feeding group: lactating cows	1.27	1.16	1.39	0.00	0.14	1.15
fitness index	0.70	0.67	0.74	0.00	0.15	1.02
floor in open air area for young stock: no open-air areas	1.86	1.69	2.05	0.00	0.14	1.13
floor in open-air area for dry cows: no open-air areas	1.38	1.26	1.50	0.00	0.15	1.10
floor in open-air area for lactating cows: no open-air areas	1.89	1.74	2.06	0.00	0.15	1.13
floor in open-air area for young stock: solid concrete	0.70	0.64	0.77	0.00	0.14	1.05
floor in open-air areas for dry cows: solid concrete	0.70	0.64	0.76	0.00	0.15	1.10

Table 11. Lameness *(continued)*

Variable	OR	CI.Low	CI.High	P-Value	R2	VIF
floor in open-air areas for lactating cows: solid concrete	0.54	0.49	0.59	0.00	0.15	1.11
floor in walkway of free-stall for young stock walkway: other	0.78	0.69	0.87	0.00	0.14	1.05
floor in walkway of free-stall for young stock: concrete slits	2.26	2.06	2.48	0.00	0.16	1.13
floor in walkway of free-stall for young stock: solid concrete	0.50	0.45	0.56	0.00	0.15	1.10
floor in walkway of free-stall for young stock: solid concrete with slits	0.71	0.58	0.86	0.00	0.14	1.06
forage dry matter intake	1.25	1.19	1.32	0.00	0.15	1.55
forage type: grass and grass products only	0.62	0.55	0.70	0.00	0.15	1.11
forage type: grass and grass products plus corn only	0.72	0.61	0.85	0.00	0.14	1.13
forage type: grass plus corn and grass products plus corn	1.17	1.01	1.36	0.04	0.14	1.03
forage type: mixed ration with concentrates	1.19	1.09	1.30	0.00	0.14	1.18
forage type: sequentially fed forages	0.64	0.58	0.70	0.00	0.15	1.24
forage type: total mixed ration	2.59	2.25	2.99	0.00	0.15	1.11
forage type: year-round silage (with corn silage)	1.42	1.27	1.59	0.00	0.14	1.06
free-stall system for dry cows: deep bed cubicle	1.14	1.04	1.24	0.00	0.14	1.05
free-stall system for dry cows: deep litter	0.53	0.45	0.62	0.00	0.14	1.04
free-stall system for dry cows: high bed cubicle	1.84	1.63	2.07	0.00	0.14	1.17
free-stall system for dry cows: other	0.67	0.58	0.76	0.00	0.14	1.05
free-stall system for lactating cows: deep bed cubicle	0.40	0.36	0.44	0.00	0.15	1.12
free-stall system for lactating cows: high bed cubicle	3.00	2.67	3.36	0.00	0.15	1.25
free-stall system for lactating cows: other	1.55	1.35	1.78	0.00	0.13	1.05
free-stall system for young stock: deep bed cubicle	0.78	0.70	0.88	0.00	0.14	1.06
free-stall system for young stock: deep litter	0.70	0.62	0.79	0.00	0.14	1.03
free-stall system for young stock: high bed cubicle	1.65	1.51	1.80	0.00	0.14	1.05
free-stall system for young stock: other	1.22	1.09	1.37	0.00	0.14	1.05
herd size	1.17	1.13	1.23	0.00	0.15	1.15
lactating cows are kept in a tie stall facility	0.69	0.58	0.82	0.00	0.14	1.05
litter in free-stall for lactating cows: chopped straw	1.63	1.48	1.79	0.00	0.14	1.16
litter in free-stall for lactating cows: long straw	0.64	0.56	0.72	0.00	0.14	1.07
litter in free-stall for lactating cows: other	0.76	0.68	0.84	0.00	0.14	1.18
main breed: Brown Swiss	0.65	0.51	0.82	0.00	0.14	5.95
main breed: Holstein	1.53	1.26	1.85	0.00	0.14	4.16
manure removal in free-stall for young stock: scraper	0.85	0.77	0.94	0.00	0.13	1.03
manure removal in free-stall for dry cows: other	0.52	0.47	0.57	0.00	0.15	1.07
manure removal in free-stall for dry cows: slits	2.17	1.97	2.38	0.00	0.15	1.10
manure removal in free-stall for lactating cows: other	0.62	0.56	0.69	0.00	0.14	1.03
manure removal in free-stall for lactating cows: scraper	1.16	1.07	1.27	0.00	0.13	1.05
manure removal in free-stall for lactating cows: slits	1.25	1.14	1.36	0.00	0.13	1.03
manure removal in free-stall for young stock: other	0.55	0.50	0.60	0.00	0.14	1.04
manure removal in free-stall for young stock: slits	2.05	1.88	2.25	0.00	0.15	1.11
manure removal: mixed forms	0.58	0.52	0.66	0.00	0.15	1.02
manure removal: slurry with perforated flooring	1.21	1.11	1.32	0.00	0.14	1.05
manure removal: slurry with solid flooring	1.13	1.04	1.23	0.00	0.14	1.03
mean content of crude fibre for dry cows	1.06	1.02	1.11	0.01	0.14	1.14
mean content of crude fibre for lactating cows	0.74	0.71	0.78	0.00	0.15	1.45
mean yearly precipitation	0.79	0.75	0.83	0.00	0.15	1.11
mean yearly relative humidity	1.08	1.04	1.13	0.00	0.14	1.15
mean yearly temperature	1.27	1.21	1.33	0.00	0.15	1.08
milking stimulation	1.20	1.09	1.32	0.00	0.14	1.12
milking take off: present	0.80	0.73	0.87	0.00	0.14	1.01
milking unit removal: present, including post-milking technology	1.53	1.36	1.74	0.00	0.14	1.09
milking vacuum	0.86	0.83	0.90	0.00	0.14	1.02
muscularity score	0.62	0.59	0.65	0.00	0.17	1.25
number of high temperature days	1.33	1.28	1.39	0.00	0.15	1.08
number of high wind days	1.19	1.14	1.24	0.00	0.15	1.14
number of low temperature days	0.88	0.84	0.92	0.00	0.14	1.05
number of milking places	1.14	1.09	1.18	0.00	0.14	1.04
pasture of dry cows	0.53	0.48	0.58	0.00	0.15	1.09
pasture of lactating cows	0.48	0.43	0.53	0.00	0.15	1.17
pasture of young stock	0.66	0.60	0.72	0.00	0.15	1.22
problematic feed quality	1.92	1.73	2.14	0.00	0.14	1.03

Table 11. Lameness *(continued)*

Variable	OR	CI.Low	CI.High	P-Value	R2	VIF
provision of concentrates: exact	0.66	0.60	0.73	0.00	0.15	1.05
provision of concentrates: total mix ration	2.55	2.21	2.94	0.00	0.15	1.09
provision of supplementary concentrate: electronic feeder	0.62	0.57	0.68	0.00	0.15	1.02
provision of supplementary concentrate: other	2.08	1.86	2.32	0.00	0.15	1.06
ration type: feedstuffs sequentially fed	0.50	0.46	0.55	0.00	0.16	1.12
ration type: partial mixed ration	1.34	1.23	1.46	0.00	0.15	1.12
ration type: total mixed ration	2.07	1.77	2.42	0.00	0.15	1.04
silo type: bunker silo	1.82	1.65	2.02	0.00	0.16	1.08
silo type: silage bales	0.53	0.47	0.60	0.00	0.15	1.13
standard deviation content of crude fibre for dry cows	1.07	1.03	1.12	0.00	0.14	1.04
standard deviation for dietary proportion of concentrates for dry cows	1.32	1.26	1.37	0.00	0.15	1.08
standard deviation for dietary proportion of concentrates for lactating cows	0.88	0.84	0.92	0.00	0.14	1.08
standard deviation of yearly relative humidity	1.08	1.03	1.12	0.00	0.14	1.08
standard deviation of yearly temperature	1.42	1.35	1.48	0.00	0.15	1.14
test-day energy corrected milk daily yield	0.84	0.80	0.89	0.00	0.15	1.20
test-day fat yield percentage	1.08	1.03	1.12	0.00	0.14	1.08
test-day fat-protein ratio	1.21	1.14	1.28	0.00	0.16	1.02
test-day protein yield percentage	0.85	0.81	0.90	0.00	0.14	1.59
test-day somatic cell count	1.21	1.16	1.26	0.00	0.15	1.14
test-day urea content	1.13	1.08	1.17	0.00	0.14	1.05
TMI	0.78	0.75	0.82	0.00	0.15	1.38
total dry matter intake	1.08	1.02	1.14	0.01	0.14	1.51
type of milking stalls: herringbone parlour	1.87	1.72	2.04	0.00	0.15	1.07
type of milking stalls: milking robot	0.72	0.64	0.82	0.00	0.14	1.10
type of milking stalls: pipe milking	0.43	0.35	0.53	0.00	0.15	1.02
type of milking stalls: side-by-side	0.86	0.75	0.98	0.03	0.14	1.03
type of milking stalls: tandem type	0.73	0.65	0.81	0.00	0.15	1.08
used forage types in diet: field forage silage, grass silage, hay, corn silage	1.15	1.03	1.28	0.01	0.14	1.05
used forage types in diet: field forage silage, grass silage, hay, corn silage, pasture	1.55	1.39	1.74	0.00	0.15	1.06
used forage types in diet: grass silage, hay	0.67	0.56	0.79	0.00	0.15	1.01
used forage types in diet: grass silage, hay, pasture	0.60	0.51	0.70	0.00	0.15	1.03
used forage types in diet: other	1.13	1.04	1.24	0.00	0.14	1.08
waist circumference	0.77	0.73	0.81	0.00	0.15	1.42
walkway floor in free-stall for dry cows: concrete slits	1.57	1.42	1.74	0.00	0.14	1.07
walkway floor in free-stall for dry cows: other	1.14	1.04	1.26	0.01	0.14	1.05
walkway floor in free-stall for dry cows: rubber mats	0.84	0.72	0.97	0.02	0.14	1.01
walkway floor in free-stall for dry cows: solid concrete with slits	0.45	0.39	0.52	0.00	0.14	1.03
walkway floor in free-stall for lactating cows: concrete slits	1.67	1.52	1.85	0.00	0.14	1.01
walkway floor in free-stall for lactating cows: rubber mats	1.14	1.02	1.27	0.02	0.13	1.02
walkway floor in free-stall for lactating cows: solid concrete with slits	0.34	0.28	0.40	0.00	0.15	1.02
young stock are kept in a tie stall facility	0.60	0.49	0.74	0.00	0.14	1.02
young stock on alpine pasture	0.57	0.52	0.62	0.00	0.15	1.19

Table 12. Odds ratios for *metritis*. ORs are obtained form the logistic regression model. Variables are listed in alphabetical order.

Variable	OR	CI.Low	CI.High	P-Value	R2	VIF
altitude	0.52	0.45	0.61	0.00	0.19	1.40
automated milking switch-off	0.53	0.39	0.73	0.00	0.15	1.12
barn design: free-stall barn	1.57	1.20	2.05	0.00	0.17	1.07
body weight	1.22	1.03	1.44	0.02	0.17	1.61
chest girth	1.31	1.13	1.52	0.00	0.17	1.29
claw trimming done by farmer	0.61	0.46	0.81	0.00	0.17	1.05
claw trimming frequency: once per year	2.45	1.75	3.44	0.00	0.17	1.30
claw trimming frequency: twice per year	0.55	0.42	0.73	0.00	0.16	1.10
diet: content of ash	0.81	0.69	0.95	0.01	0.17	1.06
diet: content of nitrogen-free extracts	1.34	1.15	1.56	0.00	0.17	1.14
diet: content of organic matter	1.23	1.05	1.45	0.01	0.17	1.06
diet: total amount of ash	0.79	0.68	0.92	0.00	0.17	1.11
diet: total amount of crude fibre	0.76	0.66	0.88	0.00	0.17	1.15
dietary proportion of concentrates for lactating cows	1.27	1.06	1.52	0.01	0.17	1.50
dietary proportion of corn silage	1.69	1.48	1.93	0.00	0.19	1.14
dietary proportion of grass silage	0.67	0.58	0.78	0.00	0.18	1.14
dietary proportion of straw	1.52	1.15	2.00	0.00	0.17	1.15
energy balance	0.84	0.73	0.97	0.02	0.17	1.27
feeding group: lactating cows	1.76	1.21	2.57	0.00	0.17	1.13
fitness index	0.70	0.61	0.81	0.00	0.18	1.03
floor in open air area for young stock: no open-air areas	1.45	1.07	1.96	0.02	0.16	1.13
floor in open-air area for dry cows: no open-air areas	4.24	2.99	6.00	0.00	0.21	1.19
floor in open-air area for lactating cows: no open-air areas	2.86	2.16	3.79	0.00	0.19	1.08
floor in open-air areas for lactating cows: solid concrete	0.33	0.24	0.45	0.00	0.19	1.02
floor in walkway of free-stall for young stock: concrete slits	1.85	1.37	2.51	0.00	0.17	1.17
floor in walkway of free-stall for young stock: solid concrete	0.47	0.33	0.66	0.00	0.17	1.22
forage type: mixed ration with concentrates	1.48	1.12	1.94	0.01	0.17	1.13
forage type: sequentially fed forages	0.53	0.40	0.70	0.00	0.19	1.15
herd size	1.28	1.12	1.46	0.00	0.17	1.25
litter in free-stall for lactating cows: chopped straw	2.61	1.87	3.65	0.00	0.17	1.07
main breed: Fleckvieh	3.52	1.82	6.79	0.00	0.17	5.34
manure removal in free-stall for dry cows: scraper	0.53	0.40	0.71	0.00	0.18	1.03
manure removal in free-stall for dry cows: slits	1.75	1.29	2.39	0.00	0.17	1.07
manure removal in free-stall for lactating cows: other	0.62	0.45	0.86	0.00	0.17	1.10
manure removal in free-stall for lactating cows: scraper	1.77	1.34	2.32	0.00	0.17	1.12
manure removal in free-stall for young stock: slits	1.62	1.20	2.17	0.00	0.17	1.13
manure removal: slurry with solid flooring	1.87	1.44	2.44	0.00	0.17	1.02
mean content of crude fibre for lactating cows	0.55	0.46	0.66	0.00	0.18	1.43
mean yearly precipitation	0.63	0.52	0.77	0.00	0.18	1.04
mean yearly relative humidity	1.28	1.09	1.50	0.00	0.17	1.13
mean yearly temperature	1.87	1.54	2.28	0.00	0.18	1.29
milking vacuum	0.77	0.71	0.84	0.00	0.16	1.01
muscularity score	0.72	0.62	0.84	0.00	0.17	1.28
number of high temperature days	1.64	1.41	1.90	0.00	0.18	1.11
number of low temperature days	0.62	0.52	0.75	0.00	0.18	1.32
number of milking places	1.35	1.23	1.50	0.00	0.17	1.12
provision of concentrates: exact	0.48	0.36	0.65	0.00	0.17	1.04
provision of supplementary concentrate: electronic feeder	0.54	0.41	0.71	0.00	0.17	1.04
ration type: feedstuffs sequentially fed	0.28	0.20	0.39	0.00	0.19	1.06
ration type: partial mixed ration	2.11	1.60	2.78	0.00	0.18	1.09
silo type: bunker silo	1.93	1.41	2.65	0.00	0.18	1.11
silo type: silage bales	0.59	0.42	0.82	0.00	0.18	1.19
standard deviation content of crude fibre for dry cows	1.21	1.08	1.35	0.00	0.17	1.05
standard deviation for dietary proportion of concentrates for dry cows	1.69	1.49	1.93	0.00	0.19	1.10
standard deviation of yearly relative humidity	1.19	1.03	1.37	0.02	0.17	1.14
standard deviation of yearly temperature	3.09	2.51	3.80	0.00	0.22	1.06
TMI	0.84	0.72	0.98	0.02	0.17	1.48
type of milking stalls: herringbone parlour	1.42	1.08	1.86	0.01	0.17	1.11
walkway floor in free-stall for lactating cows: concrete slits	1.84	1.35	2.50	0.00	0.17	1.03

Table 12. Metritis *(continued)*

Variable	OR	CI.Low	CI.High	P-Value	R2	VIF
young stock on alpine pasture	0.30	0.22	0.41	0.00	0.19	1.28

Table 13. Odds ratios for *ovarian cysts*. ORs are obtained form the logistic regression model. Variables are listed in alphabetical order.

Variable	OR	CI.Low	CI.High	P-Value	R2	VIF
altitude	0.50	0.46	0.56	0.00	0.18	1.36
automated milking switch-off	0.43	0.35	0.52	0.00	0.15	1.13
body condition score	0.87	0.79	0.95	0.00	0.15	1.06
body weight	1.26	1.14	1.40	0.00	0.15	1.58
breeding value: milk yield	1.32	1.21	1.45	0.00	0.15	1.33
claw trimming frequency: once per year	1.55	1.24	1.92	0.00	0.15	1.18
claw trimming frequency: twice per year	0.60	0.51	0.71	0.00	0.16	1.04
concentrate dry matter intake	1.34	1.21	1.49	0.00	0.15	1.33
cubicle housing system: deep bed cubicles and slatted floors	1.39	1.15	1.69	0.00	0.15	1.20
cubicle housing system: deep bed cubicles and solid floors	0.73	0.61	0.87	0.00	0.15	1.17
diet: content of ash	0.72	0.65	0.79	0.00	0.16	1.06
diet: content of crude fat	1.18	1.10	1.28	0.00	0.15	1.08
diet: content of crude fibre	0.76	0.67	0.85	0.00	0.15	1.27
diet: content of crude protein	0.90	0.81	0.99	0.03	0.15	1.17
diet: content of degradable protein	0.83	0.75	0.91	0.00	0.15	1.08
diet: content of metabolisable energy	1.35	1.20	1.51	0.00	0.15	1.27
diet: content of nitrogen-free extracts	1.38	1.26	1.52	0.00	0.16	1.11
diet: content of organic matter	1.39	1.26	1.54	0.00	0.16	1.06
diet: content of undegraded protein	1.13	1.03	1.25	0.01	0.15	1.34
diet: content of utilizable protein	1.15	1.04	1.29	0.01	0.15	1.30
diet: content ruminal nitrogen balance	0.82	0.75	0.90	0.00	0.15	1.08
diet: total amount of crude fibre	1.28	1.16	1.40	0.00	0.15	1.18
diet: total amount of crude protein	1.34	1.20	1.49	0.00	0.15	1.44
diet: total amount of degradable protein	1.27	1.14	1.40	0.00	0.15	1.34
diet: total amount of ether extracts	1.52	1.39	1.67	0.00	0.16	1.32
diet: total amount of metabolisable energy	1.69	1.52	1.89	0.00	0.16	1.49
diet: total amount of net energy	1.69	1.52	1.88	0.00	0.16	1.50
diet: total amount of nitrogen-free extracts	1.75	1.58	1.94	0.00	0.17	1.41
diet: total amount of organic matter	1.73	1.55	1.92	0.00	0.17	1.45
diet: total amount of undegraded dietary protein	1.37	1.24	1.52	0.00	0.15	1.49
diet: total amount of utilizable protein	1.61	1.45	1.80	0.00	0.16	1.50
diet: total ruminal nitrogen balance	0.86	0.79	0.94	0.00	0.15	1.09
dietary proportion of clover	1.65	1.29	2.11	0.00	0.15	1.10
dietary proportion of concentrates	1.24	1.10	1.39	0.00	0.15	1.32
dietary proportion of concentrates for dry cows	0.86	0.79	0.94	0.00	0.15	1.05
dietary proportion of concentrates for lactating cows	1.35	1.21	1.51	0.00	0.15	1.45
dietary proportion of corn silage	1.55	1.42	1.68	0.00	0.17	1.12
dietary proportion of grass silage	0.70	0.64	0.77	0.00	0.16	1.13
dietary proportion of hay	0.84	0.74	0.96	0.01	0.15	1.07
dry cows group management: separate	1.27	1.06	1.52	0.01	0.15	1.09
energy balance	1.63	1.46	1.82	0.00	0.16	1.56
farm organically managed	0.55	0.42	0.74	0.00	0.15	1.14
feeding group: lactating cows	1.69	1.36	2.10	0.00	0.15	1.09
fitness index	0.65	0.60	0.72	0.00	0.17	1.03
floor in open-air area for dry cows: no open-air areas	1.73	1.44	2.09	0.00	0.17	1.12
floor in open-air area for lactating cows: no open-air areas	1.73	1.46	2.07	0.00	0.16	1.09
floor in open-air areas for dry cows: solid concrete	0.41	0.33	0.51	0.00	0.17	1.10
floor in open-air areas for lactating cows: solid concrete	0.43	0.36	0.52	0.00	0.17	1.03
floor in walkway of free-stall for young stock: concrete slits	1.34	1.11	1.62	0.00	0.15	1.16
floor in walkway of free-stall for young stock: solid concrete	0.48	0.38	0.59	0.00	0.16	1.18
forage dry matter intake	1.36	1.23	1.51	0.00	0.15	1.39
forage type: grass plus corn and grass products plus corn	1.98	1.54	2.54	0.00	0.15	1.04
forage type: year-round silage (with corn silage)	2.29	1.90	2.78	0.00	0.16	1.05
free-stall system for dry cows: deep bed cubicle	1.26	1.06	1.50	0.01	0.15	1.02
free-stall system for young stock: high bed cubicle	1.46	1.22	1.74	0.00	0.16	1.03
herd size	1.24	1.15	1.35	0.00	0.15	1.19
litter in free-stall for lactating cows: chopped straw	1.71	1.42	2.07	0.00	0.15	1.08
litter in free-stall for lactating cows: long straw	0.67	0.52	0.86	0.00	0.15	1.06
litter in free-stall for lactating cows: other	0.62	0.49	0.79	0.00	0.15	1.16

Table 13. Ovarian cysts *(continued)*

Variable	OR	CI.Low	CI.High	P-Value	R2	VIF
main breed: Fleckvieh	2.72	1.74	4.24	0.00	0.15	6.41
manure removal in free-stall for dry cows: scraper	0.72	0.61	0.87	0.00	0.15	1.04
manure removal in free-stall for dry cows: slits	1.56	1.29	1.90	0.00	0.15	1.05
manure removal in free-stall for lactating cows: other	0.80	0.65	0.98	0.03	0.14	1.05
manure removal in free-stall for young stock: other	0.71	0.59	0.86	0.00	0.15	1.04
manure removal in free-stall for young stock: slits	2.36	1.97	2.84	0.00	0.16	1.10
manure removal: mixed forms	0.48	0.37	0.64	0.00	0.15	1.02
manure removal: slurry with perforated flooring	1.21	1.02	1.43	0.03	0.15	1.04
mean content of crude fibre for dry cows	1.17	1.08	1.26	0.00	0.15	1.04
mean content of crude fibre for lactating cows	0.70	0.63	0.78	0.00	0.16	1.35
mean yearly precipitation	0.38	0.33	0.44	0.00	0.19	1.02
mean yearly relative humidity	1.46	1.31	1.63	0.00	0.16	1.15
mean yearly temperature	1.78	1.57	2.01	0.00	0.17	1.24
milk index	1.24	1.13	1.36	0.00	0.15	1.37
milking take off: present	0.62	0.52	0.75	0.00	0.15	1.06
milking unit removal: none	1.29	1.09	1.52	0.00	0.15	1.05
milking vacuum	0.85	0.79	0.92	0.00	0.15	1.02
muscularity score	0.76	0.69	0.84	0.00	0.16	1.25
number of high temperature days	1.78	1.61	1.97	0.00	0.17	1.10
number of high wind days	1.23	1.14	1.34	0.00	0.15	1.20
number of low temperature days	0.76	0.69	0.85	0.00	0.15	1.20
number of milking places	1.18	1.09	1.27	0.00	0.15	1.09
pasture of dry cows	0.73	0.60	0.88	0.00	0.15	1.05
pasture of lactating cows	0.58	0.47	0.72	0.00	0.15	1.12
pasture of young stock	0.46	0.38	0.56	0.00	0.16	1.39
provision of concentrates: exact	0.73	0.60	0.90	0.00	0.15	1.03
provision of supplementary concentrate: other	1.60	1.28	2.01	0.00	0.15	1.08
ratio of foreign genes	0.85	0.78	0.93	0.00	0.15	1.15
ration type: feedstuffs sequentially fed	0.56	0.46	0.67	0.00	0.16	1.10
ration type: partial mixed ration	1.60	1.35	1.90	0.00	0.15	1.11
silo type: bunker silo	2.52	2.01	3.15	0.00	0.16	1.09
silo type: silage bales	0.46	0.36	0.58	0.00	0.16	1.17
standard deviation for dietary proportion of concentrates for dry cows	1.33	1.22	1.44	0.00	0.16	1.09
standard deviation of yearly precipitation	0.65	0.59	0.72	0.00	0.16	1.02
standard deviation of yearly temperature	2.40	2.13	2.70	0.00	0.19	1.04
test-day fat-protein ratio	1.17	1.04	1.31	0.01	0.14	1.01
test-day lactose content	1.20	1.07	1.34	0.00	0.15	1.26
test-day milk yield	1.70	1.52	1.90	0.00	0.17	2.00
test-day protein yield percentage	0.73	0.65	0.81	0.00	0.16	1.46
total dry matter intake	1.68	1.51	1.86	0.00	0.16	1.46
type of milking stalls: herringbone parlour	1.49	1.26	1.77	0.00	0.15	1.10
type of milking stalls: tandem type	0.73	0.59	0.89	0.00	0.15	1.06
used forage types in diet: field forage silage, grass silage, hay, corn silage	1.77	1.45	2.17	0.00	0.16	1.05
waist circumference	1.11	1.01	1.23	0.03	0.15	1.38
walkway floor in free-stall for dry cows: concrete slits	1.39	1.12	1.72	0.00	0.15	1.09
walkway floor in free-stall for dry cows: solid concrete	1.47	1.24	1.76	0.00	0.15	1.01
walkway floor in free-stall for dry cows: solid concrete with slits	0.57	0.43	0.75	0.00	0.15	1.05
walkway floor in free-stall for lactating cows: concrete slits	1.53	1.25	1.87	0.00	0.15	1.02
walkway floor in free-stall for lactating cows: rubber mats	1.45	1.17	1.81	0.00	0.15	1.05
young stock on alpine pasture	0.29	0.24	0.36	0.00	0.17	1.30

Table 14. Odds ratios for *periparturient hypocalcemia*. ORs are obtained form the logistic regression model. Variables are listed in alphabetical order.

Variable	OR	CI.Low	CI.High	P-Value	R2	VIF
altitude	0.75	0.65	0.86	0.00	0.19	1.26
beef index	0.82	0.73	0.93	0.00	0.20	1.02
body weight	1.31	1.13	1.53	0.00	0.19	1.52
breeding value: milk yield	1.28	1.11	1.47	0.00	0.19	1.43
claw trimming done by farmer	0.67	0.51	0.88	0.00	0.19	1.02
dietary proportion of corn silage	1.30	1.15	1.48	0.00	0.19	1.11
dry matter	1.25	1.05	1.48	0.01	0.24	1.36
floor in open-air areas for lactating cows: solid concrete	0.71	0.55	0.92	0.01	0.19	1.06
floor in walkway of free-stall for young stock: concrete slits	1.69	1.28	2.23	0.00	0.19	1.10
floor in walkway of free-stall for young stock: solid concrete	0.61	0.44	0.83	0.00	0.19	1.11
forage type: partial mixed ration	0.61	0.45	0.82	0.00	0.19	1.10
forage type: year-round silage (with corn silage)	2.00	1.49	2.67	0.00	0.19	1.06
free-stall system for young stock: high bed cubicle	1.56	1.20	2.03	0.00	0.19	1.03
manure removal in free-stall for dry cows: other	0.57	0.42	0.77	0.00	0.19	1.06
manure removal in free-stall for dry cows: slits	2.08	1.58	2.75	0.00	0.19	1.06
manure removal in free-stall for young stock: slits	1.46	1.11	1.91	0.01	0.19	1.08
mean content of crude fibre for lactating cows	0.71	0.60	0.83	0.00	0.19	1.36
mean yearly precipitation	0.74	0.64	0.86	0.00	0.19	1.07
mean yearly relative humidity	1.23	1.06	1.44	0.01	0.19	1.15
milk index	1.21	1.04	1.39	0.01	0.19	1.47
number of high wind days	1.20	1.07	1.34	0.00	0.19	1.13
pasture of dry cows	0.66	0.50	0.88	0.00	0.19	1.06
pasture of lactating cows	0.55	0.41	0.74	0.00	0.19	1.14
pasture of young stock	0.57	0.43	0.75	0.00	0.19	1.23
provision of concentrates: exact	0.51	0.38	0.67	0.00	0.19	1.03
provision of supplementary concentrate: electronic feeder	0.61	0.47	0.80	0.00	0.19	1.02
standard deviation for dietary proportion of concentrates for dry cows	1.25	1.10	1.42	0.00	0.19	1.08
standard deviation of yearly temperature	1.67	1.44	1.95	0.00	0.20	1.07
test-day milk yield	1.28	1.07	1.53	0.01	0.21	2.30
test-day protein yield percentage	1.26	1.09	1.45	0.00	0.20	1.60
type of milking stalls: herringbone parlour	1.82	1.40	2.35	0.00	0.20	1.09
waist circumference	1.40	1.21	1.62	0.00	0.20	1.25
walkway floor in free-stall for dry cows: concrete slits	2.16	1.62	2.89	0.00	0.18	1.08
walkway floor in free-stall for lactating cows: concrete slits	1.73	1.29	2.31	0.00	0.19	1.01
walkway floor in free-stall for lactating cows: solid concrete	1.44	1.09	1.91	0.01	0.19	1.04
young stock on alpine pasture	0.31	0.22	0.43	0.00	0.21	1.19
